# Pattern discovery and disentanglement on relational datasets

**DOI:** 10.1038/s41598-021-84869-4

**Published:** 2021-03-11

**Authors:** Andrew K. C. Wong, Pei-Yuan Zhou, Zahid A. Butt

**Affiliations:** 1 Systems Design Engineering, University of Waterloo, Waterloo, ON Canada; 2 School of Public Health and Health Systems, University of Waterloo, Waterloo, ON Canada

**Keywords:** Data mining, Machine learning, Diseases, Health care

## Abstract

Machine Learning has made impressive advances in many applications akin to human cognition for discernment. However, success has been limited in the areas of relational datasets, particularly for data with low volume, imbalanced groups, and mislabeled cases, with outputs that typically lack transparency and interpretability. The difficulties arise from the subtle overlapping and entanglement of functional and statistical relations at the source level. Hence, we have developed Pattern Discovery and Disentanglement System (PDD), which is able to discover explicit patterns from the data with various sizes, imbalanced groups, and screen out anomalies. We present herein four case studies on biomedical datasets to substantiate the efficacy of PDD. It improves prediction accuracy and facilitates transparent interpretation of discovered knowledge in an explicit representation framework PDD Knowledge Base that links the sources, the patterns, and individual patients. Hence, PDD promises broad and ground-breaking applications in genomic and biomedical machine learning.

## Introduction

Machine Learning (ML) engages in the development of theories and algorithms for building computation models to make predictions or decisions based on sample data. ML has important empirical successes on data, such as images, signals, texts and speech, with outcomes akin to human cognition and discernment. However, the interpretability of these methods is still a challenge^1,2^. When applying to relational datasets (**R**) for comprehensive clinical analysis and practice, the functional/statistical relations (reflected in Attribute-Value Associations (AVA)) overlapping with many “either-or” cases, further complicate the decision and interpretation in ML. As a result of these entanglements, ML research continue to encounter difficult problems such as (i) lacking transparency for understanding the inputs, models and outputs^2,3^; (ii) difficulty in identifying the mislabeled/anomalies^2,4^; and (iii) getting biased results when the record size is small, or the class distribution is imbalanced^5,6^.

Topol has noted in^2^ that AI focuses on accuracy improvement but provides little explanation. In the biomedical areas, this may lead to overdiagnosis in the healthy population, thus increasing the burden to health care systems instead of relieving it^7^. Current Explainable AI studies tend to focus on model explanation but not result interpretation. They are unable to spot/reveal erroneous inputs, misused features or entangled outputs. However, results interpretation is highly desired in the clinical context^7^. Methodologically, the explainability addressed in PDD attempts to meet the clinical challenges, not merely to pose a technical discourse. It intends to provide clinical results that are explainable to a clinical practitioner, comprehensible by the patients, and efficacious for selecting diagnostic characteristics, determining the therapeutic treatment of patients, and detecting rare cases from imbalanced clinical data.

To render interpretability, ML methods such as Decision Trees/Forests, Frequent Pattern Mining^8,9^ or Pattern Discovery (PD)^10^ were proposed. However, they typically produce an overwhelming number of overlapping/redundant patterns coming from entwined classes/groups^11^. These patterns are hard to partition and summarize^11–13^ for revealing precise “knowledge” inherent in the source environment, thus making interpretation difficult and lowering prediction accuracy. Recently, our bioinformatics study^14–16^ furnishes strong scientific evidence that AVA entanglement exists (even among interacting amino acids in complex protein binding environment) but can be disentangled by our new method^15^ to unveil six major statistical/functional spaces each of which reflects a specific amino-acid interacting functionality. The use of such knowledge significantly leverages prediction accuracy and renders succinct explanation.

Accordingly, a data-driven exploratory method, Pattern Discovery and Disentanglement (PDD) has been developed to discover robust/succinct patterns with statistical support and implicit functional clues that can be used to explain the underlying phenomena and augment scientific exploration as well as achieve high prediction accuracy even for rare and imbalance groups. PDD discovers deep knowledge from relational datasets. By deep knowledge^15^, we mean functions, relations and associations that are inconspicuous at the raw data level due to source entanglements but can be discovered and represented in a unified interpretable knowledgebase that links a much smaller set of explicit patterns to individual entities as well as their classes or underlying causes that begat those specific associations. This paper presents the problem-solving and explainability capabilities of PDD as applied to proteomics and disease prediction/diagnosis in support of therapeutic and prognostic evaluation to further bring forth PDD’s scientific and clinical value.

The fundamentals and theoretical development of PDD stemmed from database management^17^, statistical information theory^18,19^, pattern discovery^10,20^, pattern clustering^11^ and knowledge discovery^14–16^. We are proceeding from data to information to patterns to knowledge. Information measures and patterns are both accounting event associations deviating from random/independent default models. The former is a measure assigned to events, while the latter are tangible items extracted from the data for further analysis. To support ML tasks and explainability, we then developed pattern discovery (PD)^10,20^, pattern clustering^11^ and summarization. While PD, in principle, is able to reveal explainable patterns, yet due to their entanglement in the complex multiple source environments, it usually produced an overwhelming number of redundant/overlapping/entangled patterns defeating the purpose of explainability. This led to the development of PDD.

## Materials

**Figure 1:**
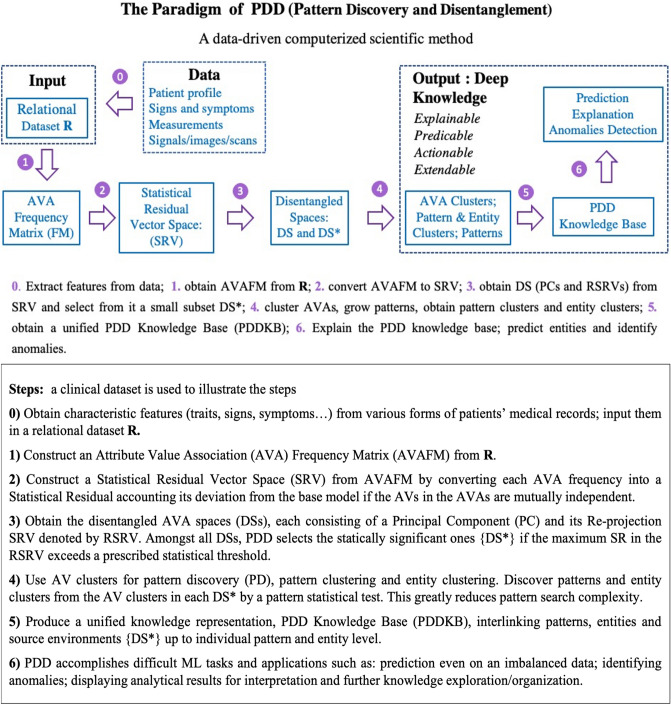
Overview of PDD. The figure describes the key ideas of the new paradigm and the algorithmic steps of PDD.

Figure 1 presents an overview of PDD, and Table 1 provides terminology descriptions with medical examples. All the detailed steps of the PDD are explained in Supplement 1 (Methodology Section), and all abbreviations are summarized in Table S1-1 in Supplement 1.

**Table 1:** Terminology.

Terminology	Brief definition	Medical examples
Pattern entanglement	Attribute Value Association (AVA) forming patterns could come from different source environments or pertain to different classes. Yet, they could be co-occurring or overlapping within entities and are hard to separate for prediction and explanation. We say that they are entangled	The AVs in the AVAs could be signs, symptoms, test results and patient's physical profile from multiple diseases or etiological causes; or mixed indicators from treatment/drug responses, etc
Disentanglement	A process to separate AVAs pertaining to different origins for they might be mixed or overlapping in the relational dataset; and to represent the disentangled AVAs in distinct statistical spaces through which patterns, pattern clusters and entity clusters can be obtained	Association of signs and symptoms from more specific pathological and etiological causes could be mixed in patient records. PDD can separate them to reveal their distinct origins; and rare cases or anomalies could be traced back to their origins related to certain disease classes/causes
Disentangled Space (DS), DSU	A Disentangled Space (DS) consists of a Principal Component (PC) and its Re-projected Statistical Residual Vector Space (RSRV) DSU is the disentangled unit represented by the ordinal number of DS, Pattern Group (PG), and Sub-PG, e.g. DSU[2 1 2] = [DS2, PGl, SubPG2]	The signs and symptoms, expressed by their statistical weights in RSRV, are more distinct, stable and specific, enhancing the statistical strength of them
Deep Knowledge	Obscured knowledge interlinking the AVA disentangled spaces (DS*), the discovered patterns and the entities. They are referred to as Deep Knowledge since they are not visualizable or recognizable at the data level	The subtle causes of a disease; manifestation of multiple disorders; misdiagnoses/mis-prognoses, best treatments identified
PDD-Knowledge Base (PDD-KB)	A unified knowledge representation consisting a Summary-KB and a Comprehensive-KB interlinking 3 parts: AVA Disentangled Space (DS*) revealing functions, discovered patterns and, entities. The knowledge base is used to support machine learning tasks, expert explanation and domain knowledge organization	DS*: disease causes, syndromes, disorders, cyberchondria; etc Pattern: associated signs-symptoms, patient’s profiles, or best treatments Entity: patients records with signs-symptoms and patient’s profiles PDDKB can link them together
EID-Intersection of an AVA	The set of entities, each contains that AVA, i.e. the intersection of entities containing that AVAs—equivalent to AVA frequency count in the dataset	Patients sharing the same group of indicators
Anomaly: outlier, and mislabeled entities	Anomalies: patterns beyond present knowledge. Two types of anomalies: (1) Outliers: entities contain no discovered patterns at certain statistical threshold but could reveal rare patterns/clues at deeper levels (2) Mislabeled: entity in classes not as labeled	Anomalies: patients found with new conditions not previously identified (1) Outliers: patient with no identified conditions of a disease complex (2) Mislabeled: Patients misdiagnosed or with misinformation in the records

The terminology table succinctly lists and briefly defines terminologies used in the paper and provides actual medical examples for each of them.

To examine the notion of pattern entanglement and disentanglement and their impact in ML and scientific applications, we designed and conducted a synthetic experiment (Supplement 2). To exemplify PDD’s capability, we used Aligned Pattern Cluster (APC) datasets, which represent local conserved function regions of protein families through the homologous aligned sites of its sequence patterns (including gaps)^21^. We treat an APC as **R** by considering each aligned site (column) as an attribute with amino acids on it as AVs (Analysis I and II). To show the efficacy of PDD in solving biomedical problems, two healthcare datasets, the Breast Cancer dataset^22^ and the Heart Disease dataset^23^ from UCI repository^24^ were used (Analysis III and Analysis IV). The details of the datasets are summarized as below.

### APC1

The first APC dataset taken from cytochrome c^21^ contains nine aligned sites (attributes) with aligned patterns from 80 samples obtained from an ensemble with imbalanced classes: 30 Mammals, 25 Plants, 20 Fungi and 5 Insects.

### APC2

Another APC was obtained from Class A Scavenger Receptor family (SR-A)^14,25^ with different function domains. It consists of 12 attributes and 95 samples taken from 5 distinct classes located in 5 different function domains. Their patterns from different functional domains were entangled, as we found later.

### Cancer dataset

It contains nine numerical cytohistological attributes with 682 cases (65.5% pertaining to Benign and the rest Malignant). To exemplify PDD’s ability to discover patterns for small/rare classes and discriminate biases/anomalies^26^, we inserted into the dataset two small transition groups—Transition1 and Transition2 (with 30 samples each, 4% of the whole data). They were stochastically generated with transitional AVs from Benign to Malignant to mimic the early stage of cancer. Figure 4a gives the quantized AVs of the transition groups. The yellow and green blocks are the majority patterns from the Benign and the transition Malignant classes respectively. The first 682 samples were taken from the original data and those from 683–712 and 713–742 were taken from Transition1 and Transition2 respectively. These small transition groups, if spotted, may help to detect the progression of cancer from early to late stage^27^.

### Heart disease dataset

The Heart Disease^23^ dataset contains 13 mixed-mode attributes (Fig. 5c) and 270 clinical records with two labeled classes: Absence or Presence of heart disease. We chose this dataset because it is mix-mode, and the AVs of both classes are very diverse.

## Result

To exemplify PDD’s data analytic capability, we employed a synthetic experiment and four analysis tasks with specific objectives using synthetic, bioinformatics and healthcare data with verifiable ground truth. In the main text, we describe the experiments and present the comparative results with their closest counterparts. In Supplement 2, we provide the entire set of experimental results with more details to exemplify the efficacy of PDD. Our experimental platform is running on a four-core intel CPU with 16 GB. The program was implemented using C# with .Net Core architecture.

*Analysis I* was designed to demonstrate the pattern discovery and disentanglement capability of PDD on an imbalanced APC dataset. Using datasets with imbalanced class distribution, it first discovered and compared the discovered patterns: (a) with or without AVA disentanglement; and (b) with or without class labels given. *Analysis II* explored PDD’s unsupervised ML capability, not relying on prior knowledge, to cluster entities coming from different function-domains/classes with noise and pattern entanglement and compared the results with those of K-means. *Analysis III* demonstrated PDD’s capability in detecting anomalies and rare groups, which were added artificially, from a cytohistological dataset (the breast cancer dataset). *Analysis IV* focused on supervised classification when anomalies are present in **R** and investigated how their identification and removal could improve classification accuracy. As for each of the above cases, we highlighted the practical aspects of PDD in solving real life genomic/healthcare problems and also its efficacy in data analytics and explainable AI.

### Analysis I: pattern disentanglement on APC1

**Figure 2:**
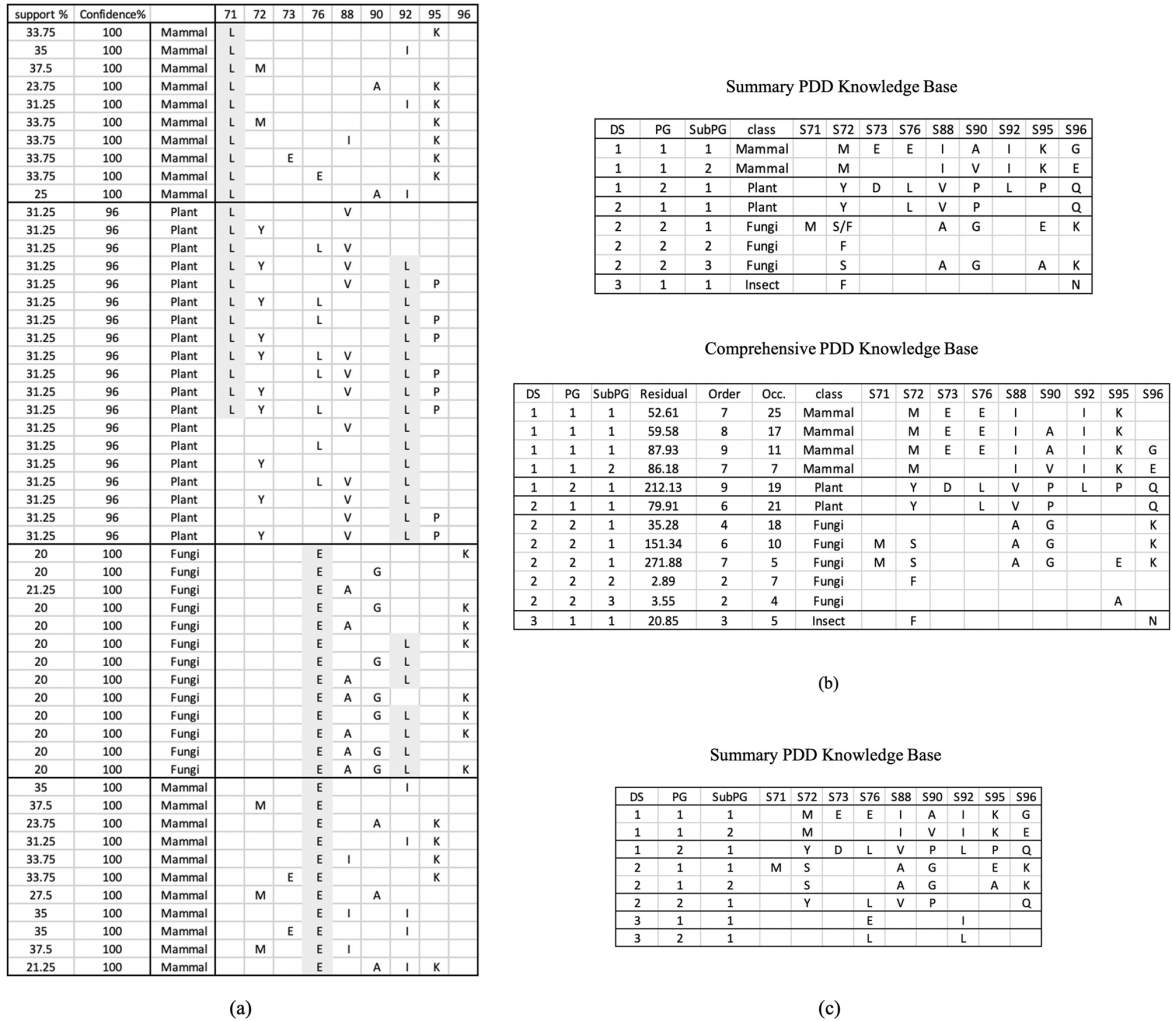
Pattern discovery and disentanglement experiment on an imbalanced APC dataset. (**a**) AVs and patterns discovered by traditional Pattern-Mining Algorithm (Apriori) from different classes are entangled as shown in shaded grey. (**b**) The summarized and comprehensive patterns discovered by PDD reside in distinct DSs associated with distinct taxonomic groups or source environments. The small “Insect” group with pattern [S72 = F, S96 = N] is found in DS3. (**c**) The results of PDD on the same set of data without class labels given produces almost identical results, indicating that PDD does not need prior knowledge to differentiate taxonomic classes in this case (see Supplement 2).

To investigate PDD’s capability in discovering patterns from entangled source environment, we applied PDD on APC1 dataset^21^, with imbalanced group distribution. We compared its results with a traditional pattern mining algorithm, Apriori^9^. Figure 2a shows partial discovered associations obtained from Apriori when setting support = 20% and confidence = 80%. From APC1, PDD discovered 12 patterns (Fig. 2b) with correct class associations covering all data, whereas Apriori^9^ (with ${\sigma }_{supp}$ = 20%, ${\sigma }_{conf}=80\%$) discovered 607 rules associating with three classes while missing the small class “Insect” since the discovered patterns by Apriori are entangled and overwhelming in number.

In Fig. 2a, note that S71 = L are found in Mammals and Plants, S92 = L found among Plants and Fungi and S76 = E among Mammals and Fungi. They are also entangled with other sub-patterns to form super-patterns. The entangled AVs may be significant for both groups or for one but not the other. For instance, S71 = L dominates Mammal and forms strong patterns with other AVs whereas it was found in half of the plant group. However, S92 = L is shared heavily by both Mammal and Fungi. These are entangled patterns. It is difficult to use their face value to infer the significance of their role in each taxonomic group.

However, after the AVA disentanglement, PDD discovered a much smaller set of 12 succinct patterns complying with all correct taxonomic classes (Fig. 2b bottom). They are summarized into 8 union patterns, referred to as summarized patterns (Fig. 2b top). Without any training process, protein segments pertaining to different functional groups were identified. Hence, PDD can discover succinct patterns in **R** for explicit interpretation.

Furthermore, Fig. 2c shows from DS3 that, without relying on class labels, PDD discovered all groups correctly, even the small insect group (with only 5 entities, 4% of the entire dataset). This validates PDD’s ability to solve the small/imbalanced class and rare pattern problems without relying on prior knowledge. In addition, in^28^, we show that PDD is able to effectively handle imbalanced class data without sampling strategies.

### Analysis II: clustering of protein segments from APC2

To show that PDD can relate pattern/entity clusters with class/functionality, we used APC2 obtained from SR-A with patterns, shown entangled later, in five diverse function domains^14,25^.

**Figure 3:**
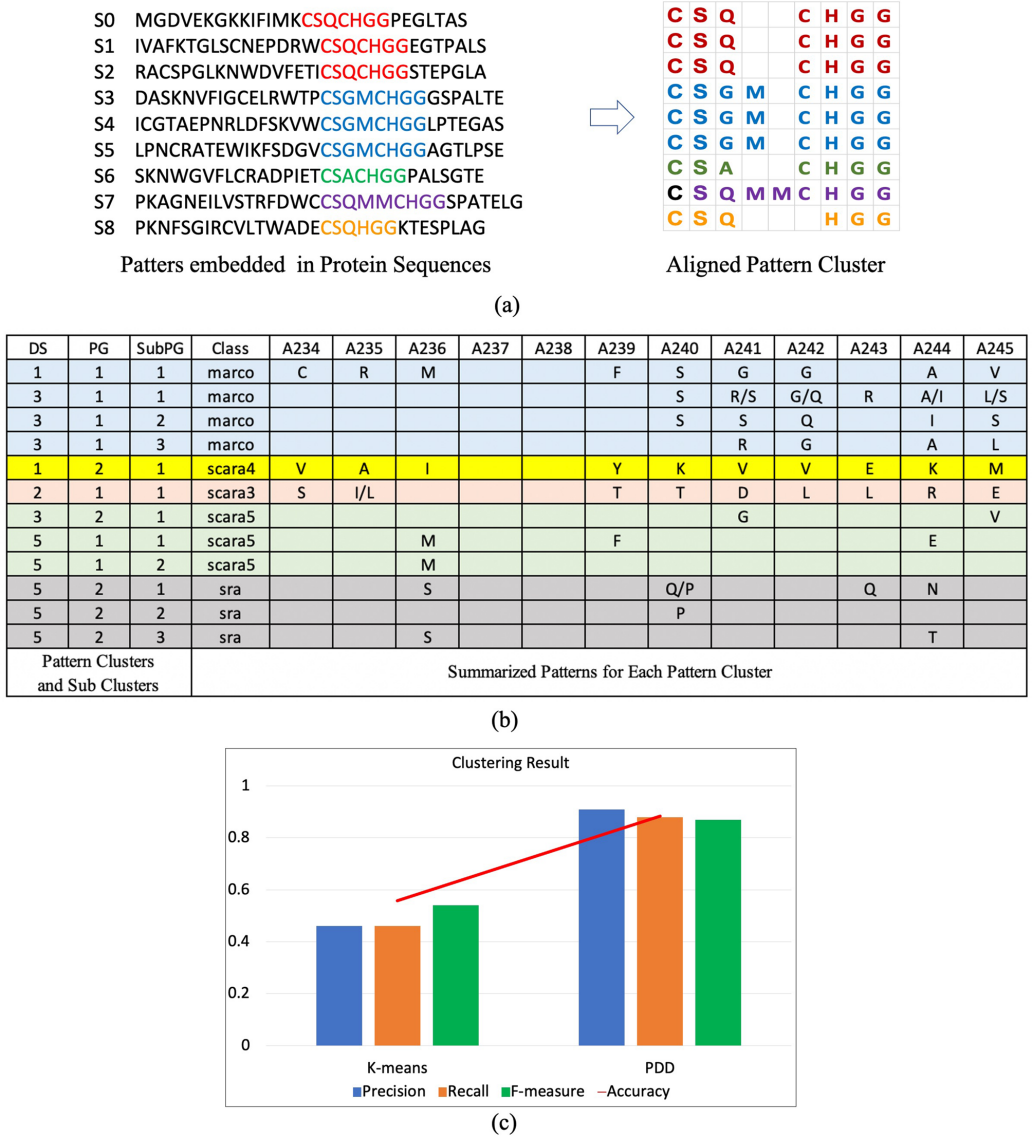
Result of pattern clustering and entity clustering on an APC representing a functional region of a protein family. (**a**) An APC obtained from a protein family. (**b**) Pattern clusters from an APC of Class A Scavenger Receptors. Patterns shown in different color shades are associated with 5 distinct classes. While K-Means could not separate *Marco* from *Scara5* and *Sra* in the collagenous domain, PDD separated *Marco* from *Scara5* in DS3 (DSU[5 1 1] and DSU[5 2 1] respectively). (**c**) Clustering scores of PDD and K-Means. PDD results are far superior to those of K-Means.

Figure 3a shows how the protein sequences are mapped into an APC dataset with the format of relational table. Then, after applying PDD on APC2, especially the disentanglement process, PDD selected four statistically significant DSs: DS1, DS2, DS3, and DS5, leaving DS4 and all the others with SR value in their RSRV below 1.96 (Fig. 3b). In this case, 12 sub-AV-clusters (SubPG) were obtained. In each AV cluster (PG), the discovered associations were from the same function domain, confirmed by the class labels placed back to **R** as references. Similar to the pattern clusters, the entities covered by the pattern clusters were also grouped into entity clusters with a similarity/overlapping check (Supplement 1). The Clustering Accuracy, F-measure, Recall and Precision^15^ were calculated based on the ground truth. The comparison results (Fig. 3c) showed that PDD outperforms K-means significantly in all scores. Tracking back to the clustering process, we found that K-means could not separate *Marco* from *Scara5* and *Sra* based just on similarity since they are in the same collagenous domain. However, PDD clearly separated *Marco* from *Scara5* in DS3 (shown by the DSU codes: [3 1 1], [3 2 1]; and *Scara5* from *Sra* in DS5 (Fig. 3b) via disentangled patterns. Hence, PDD produced pattern clusters corresponding to the correct classes as shown in distinct color shade, even those contained in the same function domains.

### Analysis III: pattern discovery, disentanglement and clustering on Wisconsin’s breast cancer dataset

**Figure 4:**
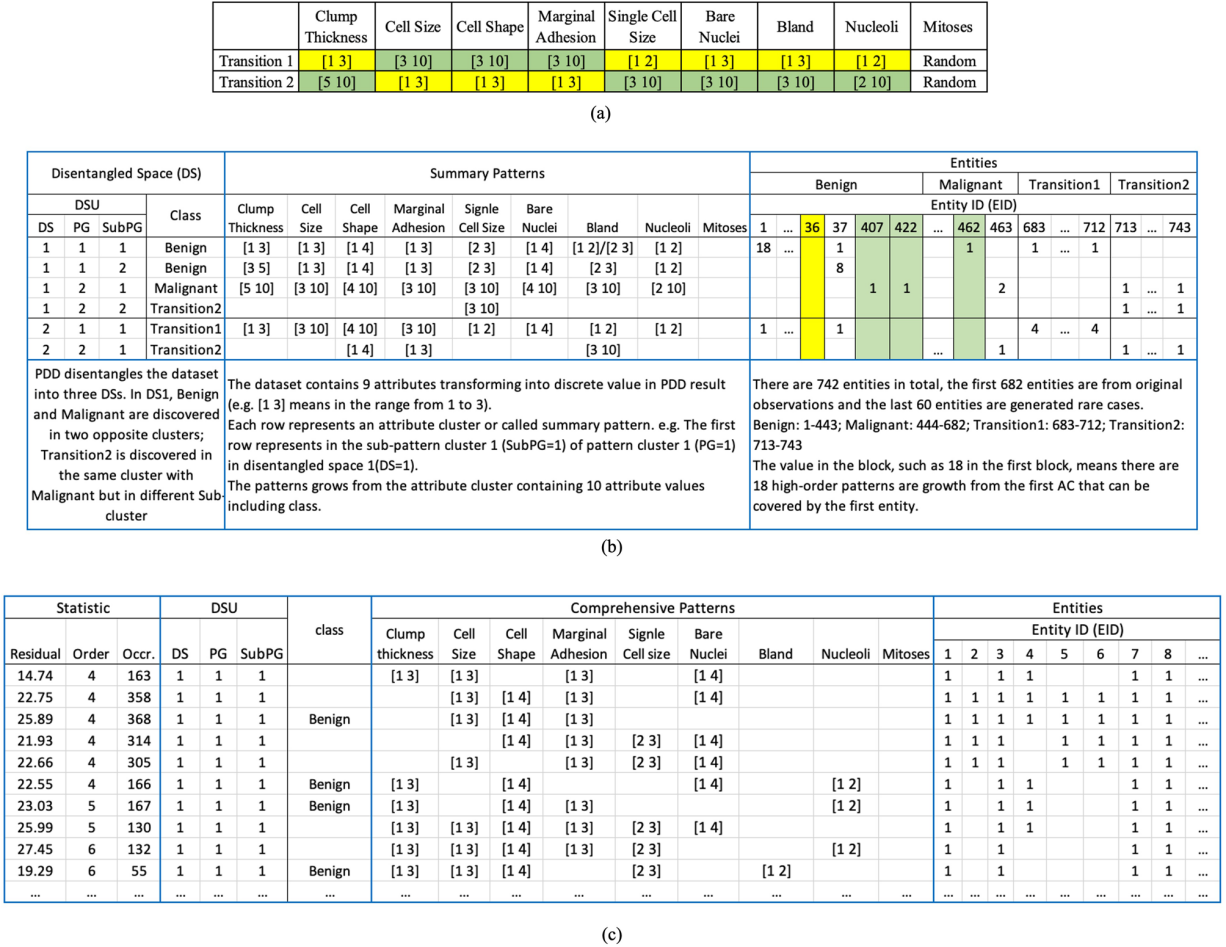
PDD knowledge base (PDDKB) for Wisconsin breast cancer dataset. (**a**) The inserted patterns for two groups of rare cases. Data quantization put each AV with small variation into the same interval. (**b**) Summary PDDKB. In the DSs, each DS Unit (DSU) (such as DSU[1 1 2] on the second row) represents SubPG2 of PG1 in DS1. The summary patterns summarize all the AV-Clusters/Patterns listed in the DSU in the Comprehensive PDDKB. For instance, the AVs in DSU[1 1 2] represent the union of all AV clusters (or patterns) found in that unit in the Comprehensive PDDKB. (**c**) Comprehensive PDDKB. Each pattern in a DSU links to a list of individual entities (denoted by ‘1’) in the column representing an entity with EID and class label (if given). In the Summary KB, the numeral on each column (like 8 associating with E37) denotes the number of patterns/pattern-clusters discovered from the DSU[1 1 2] for that entity. In the Comprehensive KB, on the same column, a numeral of “1” is displayed on the row containing a special AV cluster (or pattern) that the entity possesses.

To show the application efficacy of PDD in cytopathological research and practice, we used the Cancer dataset^22^ especially when rare cases were added artificially (Fig. 4a). Results were displayed in the PDDKB (Fig. 4b,c) interlinking DS*, patterns from each PG/SubPG and all entities in **R**.

Figure 4b shows the Summary PDDKB, containing the DS section (left), pattern section (middle) and entity section (right). They summarize all the patterns as a super-pattern (the union of the patterns) in each SubPG found in each DS* denoted by their DSU triple code. In the entity section, each column represents an individual with a distinct EID and its class label (if given). The numeral on a column and a row represents the number of patterns the entity possesses in the specific DSU containing that row. For example, the numeral 18 for entity E1 denotes that it contains 18 patterns in the DSU [1 1 1]. A summary pattern could also be treated as an AV cluster. Furthermore, as Fig. 4c shows, the Comprehensive PDDKB listed all discovered patterns. The patterns possessed by an entity were all listed in that column in the entity section. Hence, PDDKB encompasses all the integrated deep knowledge discovered from **R**. In this specific cytopathological case, note that patterns pertaining to Benign and Malignant are on the opposite side in the PC of the DS as reflected respectively from the middle digit of their DSU triple code [1 1 1] and [1 2 1]. In DS2, two inserted rare groups, Transition1 (DSU = [2 1 1]) and Transition2 (DSU = [2 2 1]), are found separated from each other. Some specific patterns as inserted (Fig. 4a) were also discovered. This demonstrates that PDD can discover rare groups, including their close or distant relation to the known groups in the PC, or differences in AVA in the RSRV, fulfilling the objectives of Analysis III.

Besides the inserted rare group, PDD can also detect the rare cases. We define two types of rare cases. One is the *Outlier,* which represents an entity that does not possess a pattern according to a prescribed statistical threshold. The other is the *Mislabeled Entity,* which only possesses patterns in one class but labeled in the original data as another class.

For example, in Fig. 4b, all 743 entities were listed in the entity section. Most of them associate with correct DSU based on their associating class labels in the Entity section. However, PDD unveiled some outliers, such as E36 shaded in yellow, because it did not possess any discovered pattern. E407 and E422, shaded in green, were identified as mislabeled entities because they were labeled as Benign in original data, but both possessed patterns in the Malignant with none in the Benign. Similarly, E462 was labeled Malignant, but possessed only patterns in the Benign. In healthcare, it is crucial if mislabeled/misdiagnosed patients can be identified earlier for therapy and treatment.

Hence, once the PPDKB is completed, simple algorithms can be used to accomplish various ML tasks such as pattern/entity clustering and supervised classification. Naturally, PDD allows and supports integrated analytics, explanation, knowledge tracking and organization to fulfil the goals of both precise data analytics and explainable AI/ML.

### Analysis IV: PDD supervised classification on heart disease dataset

**Figure 5:**
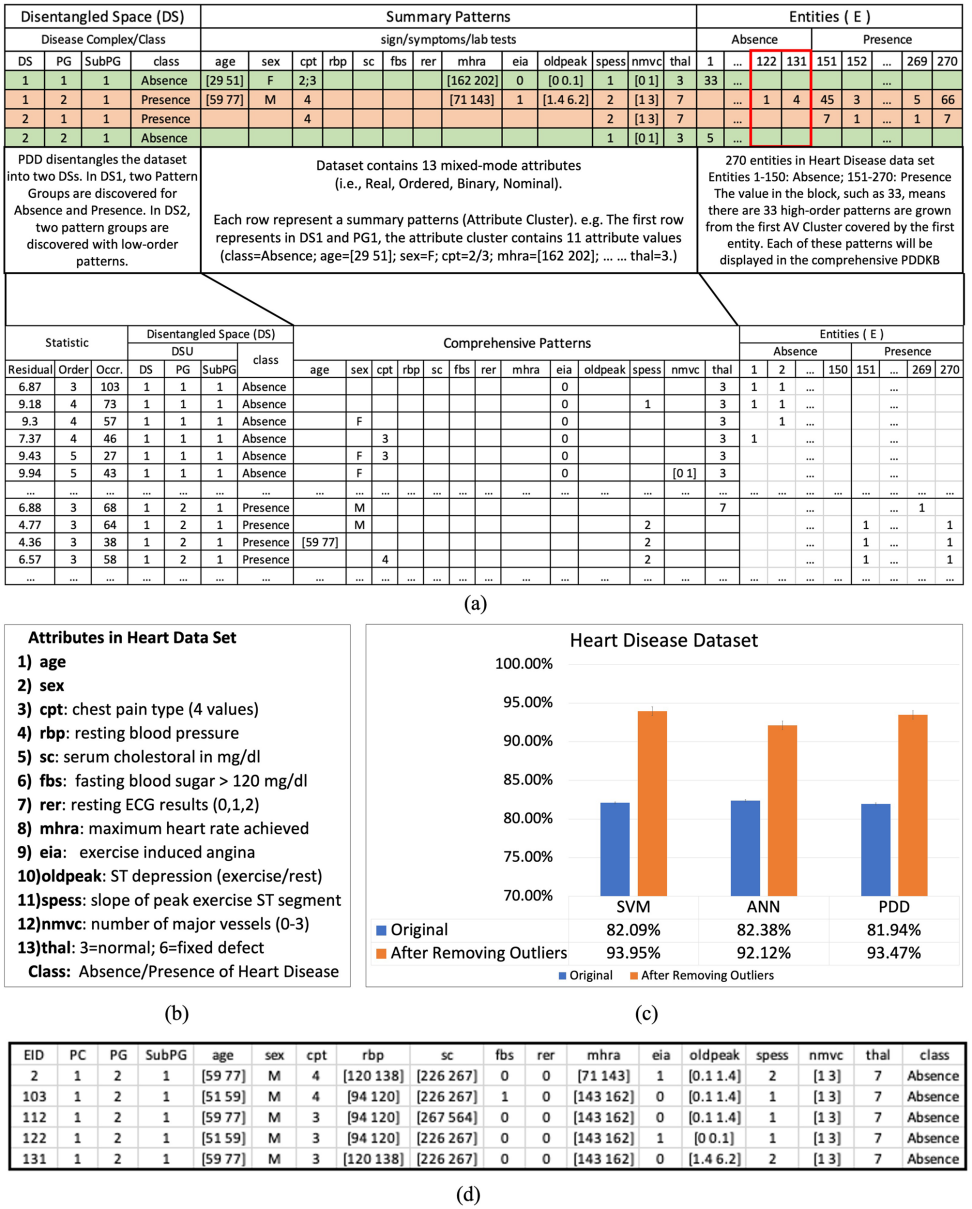
Supervised classification results of PDD, SVM and ANN on heart disease dataset. (**a**) Summary PDDKB and Comprehensive PDDKB were obtained. The blue blocks partition each into Disentangled, Pattern and Entity Spaces. The mislabeled entities E122 and E131 were discovered and displayed in the Entity Space since they were labeled as “Absence” but possessed patterns pertaining to “Presence”. (**b**) Attributes description of the Heart Disease Dataset. (**c**) Comparative rate of classification (with 80% of data for each class was selected randomly as training data and the rest (20%) as testing data by tenfold validation with variance) of PDD and other two existing ML models. After anomaly removal, the classification results of all the three models were improved approximately 10%. Such improvement cannot be realized without PDD anomaly removal and ground truth rectification process. (**d**) Entity Clustering Result showing mislabeled entities. In this case all anomalies were found among the “Absence” group but none in the “Presence” group.

As in Analysis III, we showed the significance of anomalies detection, especially in clinical practices, and presented the capability of PDD in detecting anomalies. Then in this section, by using Heart Disease dataset^23^ (Fig. 5b), we demonstrate how the classification results are improved if anomalies identified are removed from **R** before training. This indicates the rectification capability of PDD on the input and throughputs of the ML process.

In supervised learning, PDD first conducted two consistency checks before training. (A) *Outlier Check*: to identify outliers (e.g., E36 in Fig. 4b) and (B) *Abnormal Entity Check*: to identify mislabeled entities (e.g., E122 and E131 in Fig. 5a). These abnormal entities may arise from mislabelling in the given dataset or correspond to a special abnormal case or an early stage of disease although being labeled as “healthy”. Specifically, in the Heart Disease dataset^23^, parts of the results of entity clustering (Fig. 5d) showed that the mislabelled entities (e.g. E122, E131) were detected consistently with AV clustering results (Fig. 5a top) enclosed in the red boxes. Without using class labels, these two entities in the Absence group were clustered into the AV cluster DSU[1 2 1] associated with the majority Presence group as shown in Fig. 5d in which all the Presence entities labeled as Absence were listed.

To conduct supervised classification, 80% of the available data for each class was selected randomly as training data and the 20% remaining as testing data. Then the classification results of PDD were compared with those obtained from the Support Vector Machine (SVM) and Artificial Neural Network (ANN)^29^ before and after removing all detected outliers and mislabeled entities (Fig. 5c). It is obvious that after removing outliers and mislabeled cases, all classification results obtained from different algorithms were improved by approximately 10% and over.

## Discussion

As for data analytics, PDD has a significant advantage over ‘blackbox’ ML algorithms for it overcomes the major hurdles—interpretability, credibility and applicability—in ML^30^. First, as shown in Analysis I, PDD discovers patterns in the AVA disentangled spaces based on intrinsic statistically significant AVAs in different AVA Spaces without requiring explicit priori knowledge. It separated taxonomic groups including a very small Insect group with 5 samples. Second, in Analysis II, from an APC of a diverse Class A Scavenger Receptor family, patterns clusters obtained from the DSs are precisely separated corresponding to distinct functional groups. In Analysis III and Analysis IV, PDD outputs a statistical supported comprehensive and interpretable unified knowledge representation (PDDKB) containing a much smaller set of distinct and explicit patterns/pattern-clusters related to different functional sources. It interlinks patterns, source environments and individual entities, accomplishing the targeted ML tasks, and allowing biomedical knowledge interpretation, exploration and organization. In addition, the unsupervised learning results rendered superior performance of PDD to K-Means in entity clustering and anomaly detection. Finally, in Analysis IV, supervised classification comparison results show that PDD, upon the identification and removal of anomalies, could help to improve the classification performance of all three ML models by approximately 10%. Such ability of correcting ground truth is novel in ML.

In applications to real world problems as exemplified by the proteomic and medical studies, the novel capability and robustness of PDD have been empirically, statistically and functionally demonstrated. In proteomics, PDD can reveal imbalanced taxonomic classes (rare mutants) and subgroup characteristics of conserved functional domains, obtaining accurate and explicit predictive analytic results without relying on prior knowledge (Analysis I and II). In the medical data analytics, PDD furnishes clinical/statistical support, linking diagnostic patterns to the etiological origins and individual patients, with evidence explicitly displayable to medical professionals, allowing them to make further examination, testing, assessment and therapeutic decisions (Analysis III and IV). In addition, anomalies can be detected by PDD in an interpretable way rather than leaving them as undecided problematic cases. This is very important for disease diagnosis since outliers not having significant disease association and mislabeled patients in the training record attribute to lowering the diagnostic accuracy^2,4^. Hence, it can contribute significantly to early disease prediction/diagnosis, treatment, and prognosis evaluation of various conditions, particularly for depression^31^, complex neuropsychiatric disorders such as Autism Spectrum Disorders^32^ and stroke^33^.

## Conclusion

The novel theoretic concept, the efficacy of the algorithm design and the depth and breadth of the all-in-one integrated deep knowledge representation are strong evidence that PDD is a game changer in relational data analysis. It is the first AI system to discover patterns from disentangled AVA spaces, each of which relate to more specific underlying sources/causes. It represents the results in a unified representation interlinking the sources, patterns and entities together to enhance accuracy, avoid biases and render interpretability for various ML tasks and various parts of the analytical processes. Since the results which PDD obtains are robust, explicit, displayable and explainable for experts’ interpretation, question-answering and knowledge base construction, it has great potential to enhance ML and render a new form of Explainable AI^7,30,34^. It hence overcomes the limitations of current ML methods on bias, rare groups, anomalies^2–5^ and lack of transparency^3^.

In conclusion, PDD can bridge the ‘AI chasm’—the gap between creating a scientifically sound algorithm and its application to real-world problems^35^. It will play an important role in empirical and data sciences as it brings AI closer to experts with insight and accountability, meeting the scientific, economic, legal and social challenges for AI in healthcare and data analytics for the years to come.

## Supplementary information

Supplementary information.

## Data Availability

All data needed to evaluate the conclusions in the paper are present in the paper and/or the Supplementary Materials. The Heart Disease dataset and the Breast Cancer dataset are available at from the University of California Irvine Machine Learning Repository: https://archive.ics.uci.edu/ml/datasets/Statlog+(Heart); and https://archive.ics.uci.edu/ml/datasets/breast+cancer+wisconsin+(original). APCs datsets are published in our previous works, in reference^14,21,25^.
