# Supplementary material for: Pattern discovery and disentanglement on relational datasets
Source: Sci Rep. 2021 Mar 11;11:5688. doi: 10.1038/s41598-021-84869-4 (PMC7952710; doi:10.1038/s41598-021-84869-4)
Supplement: Supplementary file 1 — Supplementary information. [file 41598_2021_84869_MOESM1_ESM.pdf]

Pattern Discovery and Disentanglement on Relational Datasets

Andrew K.C. Wong[1], Pei-Yuan Zhou[1,*], Zahid A. Butt[2]
[1]Systems Design Engineering, [2]School of Public Health and Health Systems,
University of Waterloo, Waterloo, Ontario, Canada,
akcwong@uwaterloo.ca, p44zhou@uwaterloo.ca, zahid.butt@uwaterloo.ca, [*]correspondence

Supplement Note 1: Methodology

We present herein the PDD methodology applying to Relational Datasets. At the outset, we quantize the values of numeral attributes in **R** into interval values via entropy maximization [1]. PDD then accomplishes the proposed tasks in six enumerated steps, with definitions, algorithmic description and justification for each (Fig. S1-1a). Table S1-1 is a glossary that summarizes all abbreviations used in the paper.

In step 1, an AV-address Table (AT) is constructed by attaching to each AV in **R** a list of Entity Identities (EID) of the entities containing the AV. Then an AVA Frequency Matrix (AVAFM) [2] is constructed by obtaining the AVA frequency of each AV-pair. Instead of searching **R** exhaustively to look for the AV-Pair, we locate both AVs in the AT and obtain the number of the EIDs shared in both lists, i.e. the cardinality of their EID-Intersection. In Step 2, an AVA Statistical Residual Vector Space (SRV) is constructed from the AVAFM by converting each frequency into a statistical residual --- a statistical measure accounting the deviation of the observed frequency of the AVA from that if the AVs in it are independent. In Step 3, the Principal Component Decomposition (PCD) is applied on the SRV [3] to obtain a set of Principal Components (PCs) and Re-projected SRVs (RSRVs) with the same set of SRV basis vectors [3] [4]. Since in PCD, the number of DS is as large as that of AVs, we select only the statistically significant DS, denoted by DS*, if the maximum SR in its RSRV exceeds a prescribed SR threshold. In general, only a very small set of DS becomes a DS*. In Step 4, on each DS*, an AV-Clustering algorithm is

applied to obtain one or more AV clusters from the RSRV. Entities covered by an AV cluster form an entity cluster. In Step 5, a Pattern Discovery (PD) algorithm incrementally identifies the high-order patterns through confirming the pattern status by a pattern hypothesis testing of each growing AV cluster in DS* instead of through extensive search from **R**. The patterns discovered from an AV cluster/sub-cluster form a pattern group (PG)/pattern-subgroup (SubPG) respectively. Then the acquired knowledge is represented by a unified representation framework referred to as the PDD Knowledge Base (PDDKB), interlinking DS*, patterns, and all entities in **R**, in both a summarized form and a form with comprehensive details of all patterns discovered. Finally, in Step 6, difficult machine learning tasks and applications can be accomplished such as: identifying anomalies; prediction and interpreting discovered patterns, pattern and entity clusters, and supervised classification. The outputs are explicitly displayed in a knowledge representation for pattern analysis, and further knowledge exploration and organization.

Table S1-1 Glossary

**Terms**	**Description**
**RDS** or **R**	Relational Data Set
**EID; EID-I**	Entity ID of an entity in the RDS; EID-Intersection
**AV; AVA**	Attribute Value; Attribute Value Association
**AV Cluster**	Attribute Value Cluster
**SR**	Adjusted Statistical Residual
**AVAFM**	Attribute Value Association Frequency Matrix
**AVASRV (SRV)**	Attribute Value Association Adjusted Statistical Residual Vector Space
**AV-vector (*a*-vector)**	Attribute Value Vector
**PCD, PC**	Principal Component Decomposition, Principal Component
**DS; DS***	Disentangled Space; Statistically significant DS
**PG; SPG**	Pattern Group / Cluster; Sub-Pattern Group
**DSU**	Basic unit of a DS* with a triple code made up of numeral ID of DS*, PG, SPG.
**EG/EC**	Entity Group/Entity Cluster

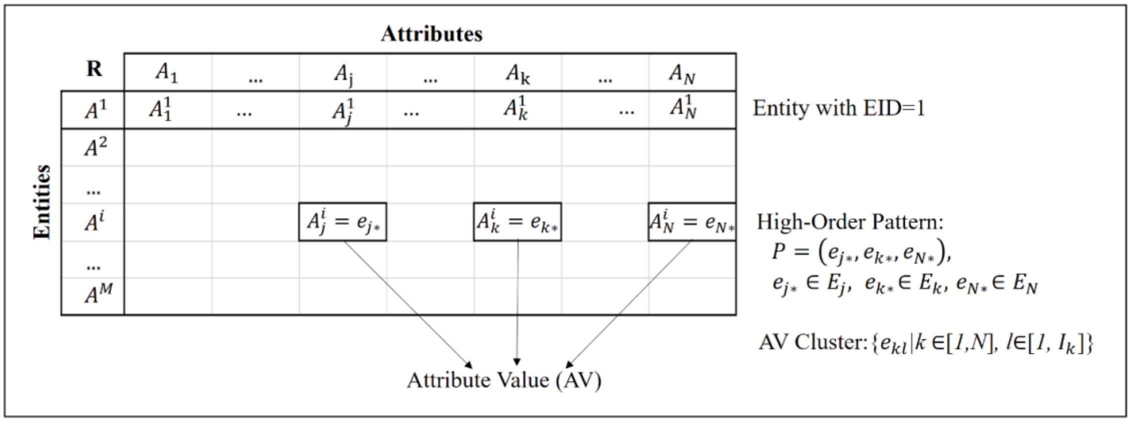

(a)

EID	class	S71	S72	S73	S76	S88	S90	S92	S95	S96
1	Mammal	L	M	E	E	I	A	I	K	T
2	Mammal	L	M	E	E	I	A	I	K	G
...	...	...	...	...	...	...	...	...	...	...
30	Mammal	L	M	E	E	I	A	I	K	G
31	Plant	L	Y	D	L	V	P	L	P	Q
32	Plant	L	Y	D	L	V	P	L	P	Q
...	...	...	...	...	...	...	...	...	...	...
55	Plant	D	V	D	Y	F	V	I	S	Q
56	Fungi	L	F	E	E	A	G	L	P	K
58	Fungi	M	S	D	E	A	G	L	P	K
...	...	...	...	...	...	...	...	...	...	...
75	Fungi	M	S	D	E	A	G	L	E	K
76	Insect	L	F	E	E	I	A	L	P	N
...	...	...	...	...	...	...	...	...	...	...
80	Insect	L	F	E	E	I	A	L	P	N

(b)

Attribute Values	Address (EID)													
class=Mammal	1	2	3	4	5	6	...	-	-	-	-	...	-	...
class=Plant	-	-	-	-	-	-	...	31	32	33	34	...	-	...
...	...	...	...	...	...	...	...	...	...	...	...	...	...	...
S71=L	1	2	3	4	5	6	...	31	32	33	34	...	-	...
S71=D	-	-	-	-	-	-	...	-	-	-	-	...	-	...
...	...	...	...	...	...	...	...	...	...	...	...	...	...	...
S96=K	-	-	-	-	-	-	...	-	-	-	-	...	-	...
S96=N	-	-	-	-	-	-	...	-	-	-	-	...	79	...

(c)

Fig. S1-1 (a) An illustration of relational dataset **R** , an example of EID, a high order pattern and an AV Cluster. Some of A_j^i in EID1 could be empty**.** (b) A part of the original APC dataset for illustration. (c) Address Table (AT) of the APC dataset.

To exemplify the entire process of the proposed algorithm, a small Aligned Pattern Cluster (APC) dataset was used. The APC dataset taken from cytochrome c [5] with 9 aligned sites (attributes) containing aligned patterns from 80 samples was obtained from an ensemble with imbalanced classes: 30 Mammals, 25 Plants, 20 Fungi and 5 Insects.

Step 0: Input and Preprocessing

Let **R** be an $N \times M$ relational dataset, where N is the number of attributes, $A_1, \ldots, A_N$ and M the number of entities A^i, $i = 1,\ldots, M$ each of which is assigned with an unique Entity ID (EID) i. Given that an attribute can take on either numerical or categorical values, **R** is in general a mixed-mode relational dataset, i.e. a set of M N-tuples of mixed-mode data (numerical / categorical). After discretizing the numerical values into quantized intervals with equal frequency, each attribute would assume a finite set of values. By treating each interval as a categorical value, **R** contains categorical values only. Let e_{ji} represent the ith value of the jth attribute in **R**. Let $E_j = \{e_{j1}, e_{j2}, \ldots e_{jI_j}\}$ be the set of all possible categorical (discrete) attribute values (AVs) of A_j, with I_j being the total number of possible values of the jth attribute. Therefore, the total number of AVs across all attributes is $\sum_{j=1}^{N} I_j$. We denote the ith entity as A^i, and the AV of the jth attribute of the ith entity as A_j^i and $A_j^i \epsilon\, E_j$. Fig. S1-1a summarizes the notations used in this section and Fig. S1-1b shows parts of the original APC dataset for illustration.

To reduce the computational complexity for Steps 2 to 6, the Entity Address Table (**AT**) is created. In the AT, for each distinct AV in **R**, it lists the Entity ID (EID) of the entities in **R** containing that AV.

***Definition 1. The Entity Address Table AT*.** **AT** is a table with $T = \sum_{j=1}^{N} I_j$ slots corresponding to all possible distinct AVs, i.e. $\cup_{j=1}^{N} E_j$, in **R**. The slot associated with an AV e_{kl} contains the list of EIDs L_{kl} of the entities containing e_{kl}, where $L_{kl} = \{i = 1, \ldots, M | A_k^i = e_{kl}\}$.

To be more specific, Fig. S1-1c shows the Address Table (AT) of the APC dataset where the EIDs are used as the addresses of the entities that contain the AV.

Step 1 and 2: Constructing Statistical Matrices

Definition 2. AVA Relative Frequency Matrix AVAFM. AVAFM is a $T \times T$ matrix of AVA relative frequencies between two AVs, say e_{ni} and $e_{n'j}$. The entry of the matrix is $f_{ni\leftrightarrow n'j} = \frac{|L_{ni} \cap L_{n'j}|}{M}$.

Through **AT**, AVAFM can be constructed using the cardinality of the EID Intersection (EDI-I) of the AV pairs, instead of through exhaustively searching and counting the AVA pairs directly from **R.** For example, let AV_i and AV_j each contains the list of entities L_{ni} and $L_{n'j}$ respectively. The number of their co-occurrence within the same entity can be obtained from $L_{ni} \cap L_{n'j}$, i.e. the intersection of their EID in the AT, instead of through searching the whole relational table. Thus, the computation complexity is greatly reduced, especially when acquiring the frequency of occurrences of high order AVAs from AV Clusters for pattern confirmation. The Adjusted Statistical Residual (SR) denoted by $SR_{ni\leftrightarrow n'j}$ between an AV pair $e_{ni\leftrightarrow n'j}$, is used to measure whether an AVA frequency (say between e_{ni} *and* $e_{n'j}$) in the FM is statistically significant or not based on Eqn (1).

$$SR_{ni\leftrightarrow n'j} = \frac{r_{ni\leftrightarrow n'j}}{\sqrt{v_{ni\leftrightarrow n'j}}} \quad (1)$$

where $r_{ni\leftrightarrow n'j}$ represents the standardized residual of $e_{ni\leftrightarrow n'j}$;

$$r_{ni\leftrightarrow n'j} = \frac{Occ(e_{ni\leftrightarrow n'j}) - Exp(e_{ni\leftrightarrow n'j})}{\sqrt{Exp(e_{ni\leftrightarrow n'j})}};$$

$v_{ni\leftrightarrow n'j}$ represents the maximum likelihood estimate of the variance of $r_{ni\leftrightarrow n'j}$ and

$$v_{ni\leftrightarrow n'j} = \mathrm{Var}(r_{ni\leftrightarrow n'j}) = (1 - \frac{|L_{ni}|}{M} * \frac{|L_{n'j}|}{M});$$

$Occ(e_{ni\leftrightarrow n'j}) = f_{ni\leftrightarrow n'j} * M$ (total number of occurrences for $A_{nk} = e_{ni}$ and $A_{n'l} = e_{n'j}$)

$Exp(e_{ni\leftrightarrow n'j}) = \frac{|L_{ni}|*|L_{n'j}|}{M}$; (expected frequency) and M is the total number of entities.

Thus, the SRV, a $T \times T$ matrix with the entry of $SR_{ni\leftrightarrow n'j}$ for each AV pair $e_{ni\leftrightarrow n'j}$, is constructed. By setting the threshold as 1.96, with confidence level = 95%, the statistically significant associations can be discovered using SR values. As an example, Fig. S1-2a shows the SRV output of the APC dataset. The blocks highlighted in green shade are the SR values exceeding 1.96.

Step 3: Disentanglement

The statistical strength and functional decomposition of SRV can reveal more definite, subtle yet significant associations that might be masked in the original frequency space. Hence, to disentangle the SRV, the Principal Component Decomposition (PCD) is applied on the SRV to obtain a set of PCs. In the SRV, we refer to the row vector associated with an AV as the *a*-vector. Thus, a set of k PCs denoted as PC = $\{PC_1, PC_2, \ldots PC_k\}$ is obtained where PC_k is a set of projections of the *a*-vectors from SRV. Then, to reveal AVAs in a disentangled manner, each PC was re-projected back to an SRV, denoted as AVA Re-projected SRV (RSRV) (using the same basis vectors). Then, certain relations (AVAs) of the *a*-vectors captured by that PC will be reflected in the corresponding RSRV. This is the essence of the SRV disentanglement (particularly if the vector is not too far from the PC axis). We adopt a consistent notation, using the subscript k in $RSRV_k$ to correspond to that in PC_k. For example, as Fig. S1-2b shows, PC_1 is the first PC revealing strong AVA of Mammal and Plant (Fig. S1-2b top) and $RSRV_1$ is their reprojection [3]. (Fig. S1-2b bottom). Same with SRV, the statistical threshold, set as 1.96, is used for revealing disentangled significant associations. In Fig. S1-2b, the strong associations, exceeded statistical threshold 1.96, are highlighted in green. We call a PC with its corresponding RSRV a Disentangled Space (DS).

SRV	class=Mammal	class=Plant	class=Fungi	class=Insect	...	S96=S	S96=E	S96=A	S96=Q	S96=Z	S96=K	S96=N
class=Mammal	0.00	0.00	0.00	0.00	...	1.77	2.70	0.29	-2.94	-1.06	-2.60	-1.37
class=Plant	0.00	0.00	0.00	0.00	...	-0.97	-1.48	-0.79	5.53	2.13	-2.37	-1.25
class=Fungi	0.00	0.00	0.00	0.00	...	-0.87	-1.32	0.71	-1.98	-0.87	6.36	-1.12
class=Insect	0.00	0.00	0.00	0.00	...	-0.43	-0.66	-0.35	-1.20	-0.43	-1.06	8.39
S71=L	0.89	0.60	-1.94	0.36	...	0.28	0.43	0.23	0.55	0.28	-2.12	0.36
S71=D	-0.61	1.23	-0.50	-0.25	...	-0.19	-0.30	-0.16	1.33	-0.19	-0.47	-0.25
S71=M	-1.94	-1.77	4.74	-0.79	...	-0.61	-0.94	-0.50	-1.70	-0.61	5.17	-0.79
S71=F	-0.61	-0.56	1.50	-0.25	...	-0.19	-0.30	-0.16	-0.54	-0.19	1.63	-0.25
S72=M	5.59	-3.06	-2.74	-1.37	...	1.77	2.70	0.29	-2.94	-1.06	-2.60	-1.37
S72=Y	-3.06	5.79	-2.10	-1.25	...	-0.97	-1.48	-0.79	5.53	2.13	-2.37	-1.25
S72=V	-0.61	1.23	-0.50	-0.25	...	-0.19	-0.30	-0.16	1.33	-0.19	-0.47	-0.25
S72=F	-2.12	-1.94	2.31	4.91	...	-0.67	-1.02	1.28	-1.86	-0.67	2.01	4.91
S95=T	1.44	-0.79	-0.71	-0.35	...	-0.27	-0.42	-0.22	-0.76	-0.27	-0.67	-0.35
S95=S	0.29	0.47	-0.71	-0.35	...	-0.27	-0.42	4.25	0.56	-0.27	-0.67	-0.35
S95=P	-3.67	3.80	-0.67	1.83	...	-1.16	-1.77	0.11	3.62	1.42	-1.09	1.83
S95=D	-0.87	-0.79	2.12	-0.35	...	-0.27	-0.42	-0.22	-0.76	-0.27	2.31	-0.35
S95=A	-1.22	-1.12	3.00	-0.50	...	-0.39	-0.59	-0.32	-1.07	-0.39	3.27	-0.50
S95=E	-1.50	-1.37	3.67	-0.61	...	-0.47	-0.72	-0.39	-1.31	-0.47	4.00	-0.61
...	...	...	...	...	...	...	...	...	...	...	...	...
S96=A	0.29	-0.79	0.71	-0.35	...	0.00	0.00	0.00	0.00	0.00	0.00	0.00
S96=Q	-2.94	5.53	-1.98	-1.20	...	0.00	0.00	0.00	0.00	0.00	0.00	0.00
S96=Z	-1.06	2.13	-0.87	-0.43	...	0.00	0.00	0.00	0.00	0.00	0.00	0.00
S96=K	-2.60	-2.37	6.36	-1.06	...	0.00	0.00	0.00	0.00	0.00	0.00	0.00
S96=N	-1.37	-1.25	-1.12	8.39	...	0.00	0.00	0.00	0.00	0.00	0.00	0.00

(a)

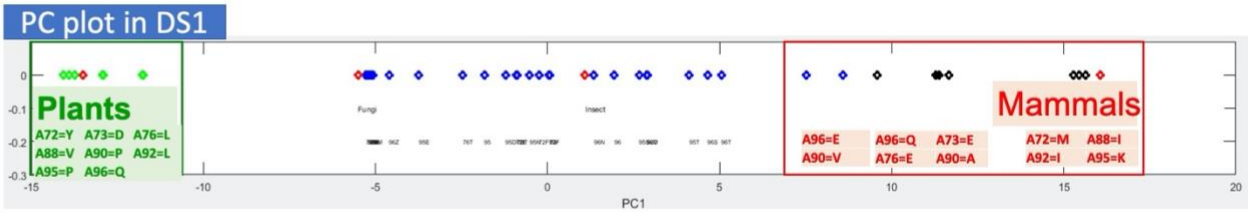

RSRV in DS1

RSRV1	S92=L	S73=D	S72=Y	S88=V	S90=P	class=Plant	S76=L	S96=Q	S95=P	...	S90=V	S96=E	S96=G	S90=A	S76=E	S73=E	S92=I	S95=K	class=Mammal	S72=M	S88=I
S88=I	-6.16	-6.00	-5.91	-5.89	-5.83	-5.76	-5.74	-5.54	-5.52	...	2.55	2.56	4.07	4.81	5.60	5.75	5.79	5.87	6.38	6.39	6.46
class=Mammal	-6.12	-5.96	-5.88	-5.85	-5.80	-5.73	-5.70	-5.51	-5.49	...	2.48	2.50	3.99	4.72	5.50	5.65	5.69	5.77	6.27	6.28	6.35
S72=M	-6.10	-5.94	-5.86	-5.83	-5.77	-5.70	-5.68	-5.49	-5.46	...	2.51	2.53	4.02	4.75	5.53	5.68	5.72	5.80	6.31	6.31	6.38
S95=K	-5.65	-5.50	-5.42	-5.39	-5.34	-5.28	-5.26	-5.08	-5.06	...	2.29	2.30	3.68	4.35	5.07	5.21	5.24	5.32	5.78	5.79	5.85
S92=I	-5.47	-5.32	-5.25	-5.22	-5.17	-5.11	-5.09	-4.91	-4.89	...	2.35	2.36	3.72	4.38	5.09	5.23	[illegible]	[illegible]	5.79	5.80	5.86
S73=E	-5.43	-5.28	-5.21	-5.18	-5.13	-5.07	-5.05	-4.87	-4.85	...	2.35	2.36	3.71	4.36	5.07	5.20	[illegible]	[illegible]	5.76	5.77	5.83
S76=E	-5.14	-5.00	-4.93	-4.90	-4.85	-4.79	-4.77	-4.60	-4.58	...	2.43	2.44	3.75	4.39	5.08	5.21	[illegible]	[illegible]	5.75	5.76	5.82
S90=A	-4.53	-4.40	-4.34	-4.32	-4.28	-4.22	-4.21	-4.06	-4.04	...	2.00	2.00	3.14	3.69	4.28	4.39	[illegible]	[illegible]	4.87	4.87	4.92
S96=G	-4.00	-3.90	-3.84	-3.82	-3.79	-3.74	-3.73	-3.60	-3.59	...	1.55	1.56	2.52	2.99	3.50	3.59	3.62	3.67	3.99	4.00	4.04
S96=E	-2.56	-2.49	-2.45	-2.44	-2.42	-2.39	-2.38	-2.30	-2.29	...	0.99	0.99	1.61	1.91	2.23	2.29	2.31	2.34	2.55	2.55	2.58
S90=V	-2.38	-2.31	-2.28	-2.27	-2.25	-2.22	-2.21	-2.13	-2.12	...	1.14	1.15	1.76	2.06	2.38	2.44	2.46	2.49	2.69	2.70	2.73
...	...	...	...	...	...	...	...	...	...	...	...	...	...	...	...	...	...	...	...	...	...
class=Fungi	1.70	1.66	1.64	1.64	1.62	1.61	1.60	1.55	1.55	...	-0.36	-0.36	-0.72	-0.89	-1.08	-1.12	-1.13	-1.15	-1.27	-1.27	-1.28
S95=P	4.92	4.79	4.72	4.70	4.65	4.59	4.57	4.41	4.39	...	-2.24	-2.25	-3.50	-4.10	-4.75	-4.88	-4.91	-4.98	-5.39	-5.40	-5.46
S76=L	4.94	4.80	4.73	4.71	4.66	4.60	4.58	4.41	4.39	...	-2.51	-2.52	-3.81	-4.44	-5.12	-5.25	-5.28	-5.35	-5.79	-5.79	-5.85
S96=Q	4.97	4.84	4.77	4.74	4.70	4.64	4.62	4.46	4.44	...	-2.22	-2.23	-3.48	-4.09	-4.74	-4.87	-4.90	-4.97	-5.39	-5.39	-5.45
S90=P	5.02	4.88	4.81	[illegible]	[illegible]	[illegible]	4.65	4.48	4.46	...	-2.55	-2.56	-3.88	-4.52	-5.21	-5.34	-5.37	-5.44	-5.88	-5.89	-5.95
S88=V	5.07	4.93	4.85	[illegible]	[illegible]	[illegible]	4.70	4.52	4.51	...	-2.58	-2.59	-3.91	-4.56	-5.25	-5.39	-5.42	-5.49	-5.94	-5.95	-6.01
class=Plant	5.11	4.97	4.90	[illegible]	[illegible]	[illegible]	4.75	4.58	4.56	...	-2.37	-2.38	-3.68	-4.31	-4.99	-5.12	-5.16	-5.23	-5.66	-5.67	-5.73
S72=Y	5.13	4.99	4.91	4.89	4.84	4.77	4.76	4.58	4.56	...	-2.56	-2.57	-3.90	-4.55	-5.25	-5.38	-5.42	-5.49	-5.94	-5.95	-6.01
S73=D	5.40	5.25	5.17	5.15	5.10	5.03	5.01	4.84	4.82	...	-2.41	-2.42	-3.77	-4.43	-5.14	-5.28	-5.31	-5.39	-5.84	-5.85	-5.91
S92=L	5.58	5.43	5.35	5.32	5.27	5.21	5.19	5.00	4.99	...	-2.44	-2.45	-3.85	-4.52	-5.25	-5.39	-5.42	-5.50	-5.97	-5.98	-6.04

AV Cluster: Mammal

AV Cluster: Plant

(b)

SRV	class = Mammal	class = Plant	class = Fungi	class = Insect
...	...	...	...	...
S72=F	-2.91	-2.53	2.89	5.50
S72=S	-2.77	-2.41	6.19	-0.92
...	...	...	...	...
S73=E	6.27	-5.70	-2.08	2.09
S73=D	-5.96	6.04	1.31	-1.99
...	...	...	...	...
S76=E	5.08	-8.22	2.17	1.69
...	...	...	...	...
S90=A	5.34	-4.05	-3.36	3.06
S90=V	3.38	-1.38	-1.84	-0.82
...	...	...	...	...
S92=L	-8.24	4.30	3.58	1.84
...	...	...	...	...
S95=A	-1.59	-1.38	3.55	-0.53
S95=E	-1.97	-1.72	4.41	-0.66
...	...	...	...	...
S96=N	-1.79	-1.56	-1.33	8.94

RSRV1	class = Mammal	class = Plant	class = Fungi	class = Insect
...	...	...	...	...
S72=F	0.12	-0.32	0.21	-0.01
S72=S	-1.61	1.19	0.62	-0.17
...	...	...	...	...
S73=E	5.65	-5.12	-1.12	0.51
S73=D	-5.96	4.97	1.66	-0.57
...	...	...	...	...
S76=E	5.50	-4.99	-1.08	0.49
...	...	...	...	...
S90=A	4.72	-4.31	-0.89	0.42
S90=V	2.48	-2.37	-0.36	0.21
...	...	...	...	...
S92=L	-6.12	5.11	1.70	-0.59
...	...	...	...	...
S95=A	-0.76	0.45	0.42	-0.09
S95=E	-1.10	0.75	0.50	-0.12
...	...	...	...	...
S96=N	0.43	-0.58	0.13	0.02

RSRV2	class = Mammal	class = Plant	class = Fungi	class = Insect
...	...	...	...	...
S72=F	-0.67	-1.29	2.14	-0.02
S72=S	-1.73	-3.42	5.60	0.00
...	...	...	...	...
S73=E	0.09	0.22	-0.31	-0.03
S73=D	0.00	0.04	-0.03	-0.03
...	...	...	...	...
S76=E	-0.90	-1.76	2.90	-0.01
...	...	...	...	...
S90=A	0.42	0.88	-1.40	-0.03
S90=V	0.30	0.64	-1.00	-0.03
...	...	...	...	...
S92=L	-0.57	-1.09	1.81	-0.02
...	...	...	...	...
S95=A	-0.91	-1.77	2.91	-0.01
S95=E	-1.35	-2.66	4.36	-0.01
...	...	...	...	...
S96=N	-0.03	-0.02	0.07	-0.03

RSRV4	class = Mammal	class = Plant	class = Fungi	class = Insect
...	...	...	...	...
S72=F	-1.48	-0.58	0.27	3.58
S72=S	0.54	0.07	0.25	-1.66
...	...	...	...	...
S73=E	-0.17	-0.16	0.26	0.19
S73=D	0.40	0.02	0.25	-1.28
...	...	...	...	...
S76=E	-0.24	-0.18	0.26	0.37
...	...	...	...	...
S90=A	-0.53	-0.27	0.26	1.11
S90=V	0.25	-0.02	0.25	-0.92
...	...	...	...	...
S92=L	-0.22	-0.18	0.26	0.31
...	...	...	...	...
S95=A	0.09	-0.08	0.26	-0.50
S95=E	0.48	0.05	0.25	-1.50
...	...	...	...	...
S96=N	-1.31	-0.53	0.27	3.15

(c)

Fig. S1-2 Illustration of the statistical matrices of the APC dataset (a) An illustration of SRV. (b) An illustration of DS* with two AV clusters in a PC. As displayed in the Re-projected SRV (RSRV), each AV in the cluster will have at least one statistically significant AVA with another AV in the cluster (significant AVAs are shaded in green in the RSRV). In the figure, the order of the AVs in the RSRV is reversed to correspond to those in the PC plot. (c) Comparison between SRV and RSRV.

To show the significance of disentanglement, we compare the SR values in the AVAs between part of the SRV and RSRVs to examine their changes after the disentanglement. For instance, in the SRV of APC, S72=F is entangled for both Fungi and Insect; S73=E is entangled for Mammal and Insect; S92=L is entangled for Plant and Fungi and so on. After the disentanglement, the AVAs associated with classes are disentangled in RSRV1, RSRV2 and RSRV4 (Fig. S1-2c). In RSRV1, the AVs associated with the class *Mammal* are disentangled from those with the class *Plant*. This is consistent with Fig. S1-2b which shows two distinct discovered AV clusters. In RSRV2, the AVs associated with the class Fungi are disentangled from those of the class Plant, and in RSRV4, the minor class, Insect, is revealed.

Since the number of DSs (PCs or RSRVs) is as large as that of AVAs and each PC is independent to the others, we then devise a DS Screening algorithm to only select the DS (denoted by DS*) that contains statistically significant SRs in their RSRV for PD. The selection of DS* is a great reduction of space complexity for PD. It is more succinct, robust and meaningful than choosing DS based on variance (eigenvalues) of the PCs. ***Algorithm 1: DS Screening*** presents the pseudocode for DS Screening.

Algorithm 1: DS Screening

Input: All RSRV= $\{RSRV_1, RSRV_2, \ldots RSRV_n\}$, each $RSRV_k = \{RSR_{ni\leftrightarrow n'j}\}$; *sig (=1.96)*
Output: Selected DS = $\{DS_1{}^*, DS_2{}^*, \ldots\}$
%For each $RSRV_k$, the task can be completed in parallel multitasking setting

Begin
  For each $RSRV_k$ in RSRV
    For each $RSR_{ni\leftrightarrow n'j}$ in $RSRV_k$
      If $RSR_{ni\leftrightarrow n'j} > sig$
        Add e_{ni} and $e_{n'j}$ in $DS_k{}^*$
      End
    End
    Add $DS_k{}^*$ in Selected DS
  Return Selected DS.
End

Step 4: AV Clustering and Entity Clustering

Once a small set of DS*s is selected, AV clusters and sub-clusters are obtained from each DS*. In addition, entity clusters and sub-clusters are generated according to their AVs discovered in the AV clusters.

Definition 3. Attribute-Value (AV) Clustering. A process finds one or more disjoint clusters in each ${DS_k}^*$ through a linear search of AV subsets from the RSRV such that each AV in the subset must have a statistically significant AVA with another AV in the subset.

The subset can be obtained incrementally in a DS*, beginning with an AV with significant AVA with another AV in the RSRV, by continual expanding the subset through linking other AV with significant AVA with any AV in the subset until no more AV can be located. The pseudo code of AV Clustering in DS* is presented in ***Algorithm 2: AV Clustering***.

To introduce sub-clusters in a hierarchical manner, AVs in each AV cluster could be agglomerated through a similarity measure between AV pairs. We use the degree of the overlapping entities in the AV pair as the measure. Consider an AV pair e_{ni} *and* $e_{n'j}$ in a DS*. We denote the similarity measure as $sim(e_{ni}, e_{n'j}) = |cov(e_{ni}) \cap cov(e_{n'j})|$, *where* $cov(e_{ni})$ and $cov(e_{n'j})$ represent the entities covered by e_{ni} and $e_{n'j}$ respectively. With the cardinality of EID-Intersection of their coverage as a distance measure, a complete-linkage hierarchical clustering algorithm is applied to obtain the clusters when a threshold which represents the lower boundary of similarity between two clusters is met. Then, to examine the deep knowledge discovered from the data (the data space), we would like to see how entities are grouped based on the AVAs in the disentangled space DS*. Like AV clusters, an entity cluster is composed of the entities containing correlated AVs which are disentangled and grouped into AV clusters. As for the AV clusters in the APC dataset

shown in Fig. S1-2b, two AV clusters in a DS* are discovered in the 1-dimensional PC plot and the 2-dimensional RSRV.

Algorithm 2: AV Clustering

Input: $\text{DS}_k{}^*$ = $\{e_{kl}|k\epsilon[1,N], l\epsilon[1,I_k]\}$, $RSRV_k$, *op*
Output: $AVCluster$ = $\{AVCluster_1, \ldots AVCluster_n\}$

% The selected significant AVAs are ranked by SR values
Initial setting: add two AVs of the first AVA in $AVCluster_1$
For each significant AVA ($e_{ni\leftrightarrow n'j}$) in $RSRV_k$
  For each existed $AVCluster_k$
    If e_{ni} (or $e_{n'j}$) has been in $AVCluster_k$
      add $e_{n'j}$ (or e_{ni}) in $AVCluster_k$
    End
  End
  If e_{ni} *and* $e_{n'j}$ are not in any existed $AVCluster_k$
    create $AVCluster_{k+1}$
    add e_{ni} *and* $e_{n'j}$ to $AVCluster_{k+1}$
  End
End
Return $AVCluster$ =$\{AVCluster_1, \ldots AVCluster_n\}$

Definition 4. Entity Clustering (EC) in Disentangled Spaces.

EC is a process to assign each entity in **R** to an AV Cluster in DS* by maximizing the number of AVs it shares with the entity, i.e. the number of AVs in that AV cluster and that entity. Its pseudo code is given in ***Algorithm 3:Entity Clustering***.

Step 5: Discovery of High-Order Patterns, Pattern Clusters and Pattern Sub-Clusters

After obtaining AV clusters and sub-clusters in different DSs*, a pattern discovery procedure applying statistical pattern hypothesis testing on each AV cluster/sub-cluster is used to discover high-order patterns in each DS*. Fig. S1-1a gives an example of three AVs co-occurring on the same entities. If their frequency of co-occurrences on same entities in **R** exceeds the statistical threshold, they become a pattern [1].

Algorithm 3: Entity Clustering

Input:
$\boldsymbol{AVCluster} = \{AVCluster_1, \ldots AVCluster_{n+1}\}$
$\mathbf{R}$ $(A^i = \{A^i_n | A^i_n \epsilon \{e_{n1}, e_{n2}, \ldots e_{nI_n}\}\}$
Output: $EC = \{EC_1, \ldots EC_{n+1}\}$
Procedure ***EntityClustering***

Begin
  For each entity, A^i, in R
    For each $AVCluster_k$
      Sharing = $A^i \cap AVCluster_k$
    End
  Assign A^i into EC_k by maximizing *sharing*
  End

  Return $EC = \{EC_1, \ldots EC_{n+1}\}$

$$EC_k = \{A^i | A^i = [A^i_1, \ldots A^i_N],\ A^i_n \epsilon \{e_{n1}, e_{n2}, \ldots e_{nI_n}\}\}$$

End

Definition 5. High-Order Pattern. A high-order pattern P_j consists of a subset of AVs with size≥2, such that the frequency of their co-occurrences on the same entities in **R** deviates significantly from the random default model, i.e. that the distribution of AVs is equi-probable and they are independent in their occurrences.

Fig. S1-1a shows an example of high-order patterns. In PD, *Adjusted Residual* for a candidate pattern derived from the frequency of co-occurrences of a high order AVA is used in the hypothesis test for determining the statistically significant pattern. The SR for a pattern P_j is derived as

$$SR(P_j) = \frac{R(P_j)}{\sqrt{V(P_j)}} \tag{2}$$

where $R(P_j) = \frac{Occ(P_j) - Exp(P_j)}{\sqrt{Exp(P_j)}}$ is the standard residual or Pearson Residuals, $Occ(P_j) = |\bigcap_{e_{nj} \epsilon E_n} L_{nj}|$ is the frequency of its co-occurrences on the same entities; and $Exp(P_j) =$

$M \prod_{e_{nj} \epsilon E_n} \frac{Occ(j)}{M}$ is the expected occurrences on the same entities, and $V(P_j) = 1 - \prod_{e_{nj} \epsilon E_n} \frac{Occ(P_j)}{M}$ is the standard deviation of all the residuals, M is the number of tuples of **R.**

In order to keep the discovered patterns non-redundant, we only accept delta-closed patterns [6] [7] in the PD process. We adopt the processes reported in [8], which also provides the definition of sub-patterns, super-patterns and delta-closed patterns, for pattern pruning.

In summary, PD on DS* is a process of identifying high order patterns from the AV clusters which are incrementally growing through the AV association (Definition 3) and through the pattern status confirmation hypothesis testing (Definition 5). In another words, it can be considered as a pattern growing process from the second-order patterns $P_i = \{e_{ni}, e_{n'j}\}$ found in the DS * to third and higher order patterns using the significant AVA information (SR) tabulated in the RSRV. There might be more than one pattern identified in the AV cluster/sub-cluster. The discovered patterns within an AV cluster/sub-cluster will constitute a pattern cluster (PG).

The patterns discovered by PDD in the DS* (AV clusters) naturally become pattern clusters; and the entity clusters generated from the AV clusters will relate the entities directly with the orthogonal source environment captured in the DS*. Hence, the output is an all-in-one representation referred to as a PDD Knowledgebase (PDDKB) which links the source environment, patterns and entities altogether for the ease of executing various posteriori data analytic tasks and interpretation.

As for the APC dataset shown in Fig. S1-3, 12 high-order patterns discovered and displayed in the Comprehensive PDDKB comply with all correct taxonomic classes. The Summary PDDKB summarized the AV clusters. In this case, even without any training process, protein segments pertaining to different functional groups could still be separated and identified by PDD.

Summary PDD Knowledge Base

DS	PG	SubPG	class	S71	S72	S73	S76	S88	S90	S92	S95	S96
1	1	1	Mammal		M	E	E	I	A	I	K	G
1	1	2	Mammal		M			I	V	I	K	E
1	2	1	Plant		Y	D	L	V	P	L	P	Q
2	1	1	Plant		Y		L	V	P			Q
2	2	1	Fungi	M	S/F			A	G		E	K
2	2	2	Fungi		F							
2	2	3	Fungi		S			A	G		A	K
3	1	1	Insect		F							N

Comprehensive PDD Knowledge Base

DS	PG	SubPG	Residual	Order	Occ.	class	S71	S72	S73	S76	S88	S90	S92	S95	S96
1	1	1	52.61	7	25	Mammal		M	E	E	I		I	K	
1	1	1	59.58	8	17	Mammal		M	E	E	I	A	I	K	
1	1	1	87.93	9	11	Mammal		M	E	E	I	A	I	K	G
1	1	2	86.18	7	7	Mammal		M			I	V	I	K	E
1	2	1	212.13	9	19	Plant		Y	D	L	V	P	L	P	Q
2	1	1	79.91	6	21	Plant		Y		L	V	P			Q
2	2	1	35.28	4	18	Fungi					A	G			K
2	2	1	151.34	6	10	Fungi	M	S			A	G			K
2	2	1	271.88	7	5	Fungi	M	S			A	G		E	K
2	2	2	2.89	2	7	Fungi		F							
2	2	3	3.55	2	4	Fungi								A	
3	1	1	20.85	3	5	Insect		F							N

Fig. S1-3: High-order Patterns obtained from the APC Dataset. The Summary PDDKB and Comprehensive PDDKB are constructed for presenting the discovered knowledge.

Step 6: Deep Knowledge Representation for Explanation and Prediction

If the class labels are given, the discovered patterns that are associated with class could be used for supervised learning using associative classification algorithms [9]. The classification accuracy is assessed by taking the class labels as the ground truth. If the class labels are unavailable or not given in **R**, both the pattern clusters and the entity clusters are the unsupervised outcomes of PDD. Their accuracy can be assessed after assigning the class labels back to the corresponding entities. As shown in the main text, most results from cases with or without class labels given are almost identical. Such succinct phenomena are observed in all the four case studies. It gives very strong support that the patterns discovered in DS* by PDD are much more distinctly associating with the disentangled sources reflecting the inherent characteristics of different classes/groups. Thus, without relying on prior knowledge, PDD can interlink AV clusters (function association), patterns, and entities together in a PDD Knowledge Base (PDDKB) to enhance supervised (with class labels), unsupervised (without class labels) and semi-supervised ML.

Since PDD works with/without class labels [4] [2], it is able to address ML in a more general setting, including discovering rare events/patterns in DS* and solving the imbalanced class, bias and mislabeling problems. Due to the transparency of its process, contents of its throughput and output, PDD can reveal established knowledge and/or new findings inherent in the throughput/output data assisted by experts and/or supporting evidence and experimental verification. In supervised classification, its ability to identify anomalies (outliers/mislabelled) from **R** before training is novel in ML. Its analytical capability and explainability extend to the input so as to validate and correct the ground truth before its use for training the classifier.

Parameter Setting

To grow high-order patterns in a DS* from their RSRVs, we need only to specify two parameters in PDD: 1) statistical significance threshold *sig*; and 2) delta tolerance factor $\boldsymbol{\delta}$ for pruning patterns; *sig* $SR(P_j)$ is used to assess the pattern P_j. In statistic, setting *sig* to be 1.96, corresponding to the p-value of 0.025 [10] is a common practice. The parameter δ is the sufficient fraction for a pattern to be considered as being mostly covered by its super-pattern [6] [8]. We choose $\delta = 0.8$ as a good practice. The *overlap* is an optional parameter used to evaluate the density of pattern sub-clusters with [0,1]. The upper bound, 1, corresponds to the patterns in the same cluster covering the same entities, while the lower bound, 0, corresponding to no sub-cluster are constructed. For the *overlap=n* (0<n<1) corresponds to the overlapping percentage of the entities covered by the patterns in the same SubPG, not larger than $\mathrm{n} \times 100\%$. We set overlap=0.5 as a reasonable choice.

Time Complexity Analysis

The time-complexity analysis of PDD, our other recent work [3] [2] and traditional PD [1] [8] for their major procedures is given as below.

1) SRV Construction: when AT is used for SRV construction, for an N x M matrix **R**, the time-complexity of PDD is O(MN) compared with $O((MN)^2$,of our previous method [1].

2) Decomposition: SRV is an $n \times n$ matrix where n is the number of AVs. The time complexities of decomposition and reprojection using PCD are $O(n^3)$ [11] and $O(n^2)$ respectively. Hence, the total time complexity of obtaining DS is $O(n^3)$+$O(n^2)$. However, it can be reduced to $O(n^2)$ [12] when a distributed implementation of stochastic PCD is applied.

3) DS* Screening: for each DS, we search its RSRV for an SR exceeding the statistical threshold on the matrix. Since there are n of them, the complexity is $O(n^2)$.

4) PD on each DS*: The complexity of PD is exponential [13]. However, PDD reduces the number of candidates dramatically from all entities in **R** to a very small significant space DS*, so the time complexity is also reduced to $O(2^c)$, where c is the number of candidate AVs in DS*, which is very small in comparison with n.

In addition, the study [8] [14] showed great complexity challenges in the pattern post-analysis, from fundamental concepts and algorithmic approaches, to existing data mining discipline. First, the computation complexity of PD is exponential $O(2^N)$, and that of the pattern pruning is $O(p^2)$, where p depends on the number of patterns. p may be huge since the mined patterns are always redundant [8]. Even after pruning the mined patterns into a small number p', the complexity of pattern clustering algorithm and K-means, is $O(p'^3)$, where p>>n and p'>>n.

Therefore, instead of discovering, clustering and summarizing patterns with high complexity by traditional approaches [8] [14], PDD can obtain disentangled high-order patterns, PGs, SubPGs, ECs in an all-in-one step with low complexity. Since DS* is independent of each other, the PDD

computational process can be executed in a parallel multitasking setting, further improving the speed of the entire process.

Supplement Note 2: Materials and Additional Experimental Result

Materials

In this supplement, we will go through the parts briefly described in the main text briefly but will furnish more comprehensive explanation and exposition on the detailed parts of the experiments.

Synthetic Dataset: We generated a 3000 x 16 matrix with the first column as class label and other as attributes with character values stochastically generated from a uniform distribution. We then embedded patterns of three different classes C_1, C_2, and C_3 for the first ten attributes and randomly embedded character values for the other five attributes. We used A1A to represent character value A of the attribute A1, and A3E/F to represent value E or F of attribute A3 and so forth for the first ten attributes in Fig. S2-1a. For the last five attributes, we put in randomly selected characters from {“O”, “P”, “Q”} as their values. The patterns implanted in **R** are given in Fig. S2-1a. Note that A1A, A2C, A3E/F are entangled (overlapping) for C1 and C2; A4H, A5M, A6A/B are entangled in C1 and C3; A7I, A8J, A9G/K are entangled in C2 and C3.

APC Data Set 1 (APC1): A small APC contains nine attributes and 80 aligned patterns obtained from samples with imbalanced class size (Mammals 30, Plants 25, Fungi 20 and Insects 5) [5].

APC Data Set 2 (APC2): A larger APC contains 95 samples and 12 attributes obtained from a protein family known as class A scavenger receptor [15]. This receptor has five distinct classes (Marco, SRA, Scara3, Scara4, and Scara5) locating in five different function domains: Cytoplasmic, Collagenous, Transmembrane, a-helical and coiled-coil motifs.

Breast Cancer Wisconsin Data Set (Cancer): The Breast Cancer Wisconsin dataset [16] is a health care benchmark dataset taken from UCI repository [17], which is a classical dataset with 682 cases

for discriminating the instances of two possible classes: Benign (distribution=65.5%) and Malignant (distribution=34.5%).

Heart Disease Data Set (Heart): Heart Disease [18] dataset is a health care benchmark dataset from UCI repository [17], which contains 270 clinical records with 13 mixed-mode attributes in two possible classes: Absence or Presence (of heart disease).

Analysis I: Disentangled Pattern Discovery on Synthetic Data and Others

Since PDD is built upon pattern discovery and disentanglement, we first design a synthetic experiment to exemplify such a unique and novel concept with strong statistical backing. We then supplement Analysis I in the main text with results from Cancer [16] and Heart [18] dataset and use these results to exemplify PDD's capability in discovering rare patterns.

Disentanglement Results of PDD on Synthetic Data

In the synthetic data, we implanted the same sub-pattern in two different classes, indicating that it is common to and overlapping both classes. Moreover, the sub-pattern also co-occurs or overlaps with other sub-patterns of the same or different classes. Hence, in a certain sense, patterns from different groups and even from the same entities are entangled. They are related to different classes yet difficult to separate. As our experimental results showed, without disentanglement, traditional methods, like Apriori [19] and PD [1] [14], produces far too many redundant patterns (Fig. S2-1b and c) but fails to render succinct pattern clusters associating with the implanted classes.

The results of Apriori [19] varied depending on the support or confidence set. For (supp=20%, con=80%), it discovered 254 patterns associating with C2 and C3 but none with C1, since many of the C1 patterns overlap with C2 and C3 as partially shown in Fig.S2-1b. For support=10%, 4041 patterns associating with various classes were discovered. Many were associated with AV's

contained in the random attributes (A11 to A15). When using Apriori, we have to set the "Consequent" with class labels given (Fig. 2-1a).

When PD [1], with significant level = 1.96 and the maximum order = 10, was applied, a large number (12,312) of patterns (up to order 10) were discovered. Fig. S2-1c shows the combinatorial nature of the patterns discovered. Though we could rank the patterns, yet it was difficult to derive succinct explanation. When pattern clustering [14] was applied, three large pattern clusters were obtained (Fig. S2-1d). The patterns were entangled in each cluster. Furthermore, both the space and time complexity of PD were high.

When PDD was applied to **R,** a much smaller set of patterns were obtained from the disentangled spaces (PG/SubPG) without relying on class information. PDD discovered 43 patterns, 22 of them pertain to distinct classes as shown in the comprehensive PDDKB (Fig. S2-1e). The disentangled patterns were discovered via the AV Clusters found in the DS*s. From the Comprehensive PDDKB, disentangled patterns pertaining to C1 and C2 were found in PSG1 of DSU[1 1 1] in DS1 and PSG1 of DSU[2 1 1] in DS2 respectively. Patterns pertaining to C3 were found in both PG2 of DSU[1 2 1] and PG2 of DSU[2 2 1] respectively. Since PDD discovers patterns not based on classed labels, patterns pertaining to different classes can be found in the same DS if they share strong sub-pattern(s). For example, a pattern in DSU[2 2 1] consisting of AVAs [A5M, A7D, A8F] pertaining to C1 was discovered in the same DS containing other patterns pertaining to C3. However, the other sub-pattern of C1 consisting of [A3F, A4H, A5M] sharing A5M is common to a sub-pattern of C1 in DSU[1 1 1]. Nevertheless, each pattern, which might consist of sub-patterns found in other classes, was still found associated with one distinct class. Hence, in clinical application, PDD is able to discover subset of signs-and-symptoms shared by different disease complexes. Furthermore, unlike cases in Apriori (Fig. S2-1d), no pattern discovered by PDD was

associated with A10-A15 since they were random attributes containing only noise. When we checked the entities clustering result, we found all those entities labeled as C1 were clustered into Entity Group (EG) within DSU[1 1 1] and all those labeled as C2 and C3 were clustered within DSU[2 1 1] and DSU[1 2 1] respectively.

Classes	Attribute Values are Significant Associated with Class Label
C1	A1A, A2C, A3E/F, A4H, A5M, A6A/B, A7D, A8F, A9G, A10N/L
C2	A1A, A2C, A3E/F, A4G, A5N, A6A, A7I, A8J, A9K/G, A10N/L,
C3	A1B, A2D, A3F, A4H, A5M, A6A/B, A7I, A8J, A9K/G, A10N/L,

(a)

Consequent	Antecedent	Support %	Confidence %
Class = C2	A5 = N	33.333	100
Class = C2	A4 = G	33.333	100
Class = C3	A1 = B	33.333	100
Class = C1	A7 = D	33.333	100
Class = C1	A8 = F	33.333	100
Class = C3	A2 = D	33.333	100
Class = C2	A5 = N and A4 = G	33.333	100
Class = C2	A5 = N and A2 = C	33.333	100
Class = C2	A5 = N and A1 = A	33.333	100
Class = C2	A5 = N and A7 = I	33.333	100
Class = C2	A5 = N and A8 = J	33.333	100
…	…	…	…
Class = C2	A14 = P and A5 = N	10.333	100
Class = C2	A14 = P and A4 = G	10.333	100
Class = C1	A13 = Q and A7 = D	11.333	100
…	…	…	…

(b)

Index	Residual	Prob.	Occurence	Order	A1	A2	A3	A4	A5	A6	A7	A8	A9	A10	A11	A12	A13	A14	A15	Class
0	32.35	0.33	1000	5		C		H	M		D	F								
1	32.35	0.33	1000	5	B		F	H	M											C3
2	32.35	0.33	1000	5	B			H			I	J								C3
3	32.35	0.33	1000	5	B	D			M		I	J								
4	32.35	0.33	1000	5	A	C		H			D	F								
...	...	...	...	...	...	...	...	...	...	...	...	...	...	...	...	...	...	...	...	...
12310	1.96	0.05	160	6		C		H	M				G	N		P				
12311	1.96	0.05	160	6	A	C			M				G	N		P				

(c)

Pattern Cluster 1																															
A1		A2		A3		A4		A5		A6		A7		A8		A9		A10		A11		A12		A13		A14		A15		Class	
A	0.71	C	0.71	E	0.36	H	0.64	M	0.64	B	0.27	D	0.36	F	0.36	G	0.72	N	0.50	P	0.27	Q	0.35	P	0.37	P	0.29	Q	0.41	C1	0.36
B	0.29	D	0.29	F	0.64	G	0.36	N	0.36	A	0.73	I	0.64	J	0.64	K	0.28	L	0.50	Q	0.44	O	0.34	Q	0.30	Q	0.37	O	0.30	C2	0.36
																				O	0.28	P	0.31	O	0.32	O	0.34	P	0.30	C3	0.29
Pattern Cluster 2																															
A1		A2		A3		A4		A5		A6		A7		A8		A9		A10		A11		A12		A13		A14		A15		Class	
A	0.64	C	0.64	E	0.30	H	0.64	M	0.64	B	0.25	D	0.29	F	0.29	G	0.68	N	0.47	P	0.26	Q	0.31	P	0.36	P	0.31	Q	0.40	C1	0.29
B	0.36	D	0.36	F	0.70	G	0.36	N	0.36	A	0.75	I	0.71	J	0.71	K	0.32	L	0.53	Q	0.42	O	0.33	Q	0.30	Q	0.38	O	0.31	C2	0.36
Pattern Cluster 3																															
A1		A2		A3		A4		A5		A6		A7		A8		A9		A10		A11		A12		A13		A14		A15		Class	
A	0.64	C	0.64	E	0.33	H	0.71	M	0.71	B	0.30	D	0.36	F	0.36	G	0.72	N	0.50	P	0.27	Q	0.32	P	0.40	P	0.29	Q	0.36	C1	0.36
B	0.36	D	0.36	F	0.67	G	0.29	N	0.29	A	0.70	I	0.64	J	0.64	K	0.28	L	0.50	Q	0.42	O	0.36	Q	0.32	Q	0.38	O	0.31	C2	0.29

(d)

Summary PDD Knowledge Base

DS	PG	SubPG				A1	A2	A3	A4	A5	A6	A7	A8	A9	A10	A11	A12	A13	A14	A15	Class
1	1	1				A	C	E	H	M	B	D	F	G							C1
1	2	1				B	D	F			A	I	J	K							C3/C2
1	2	2							G	N		I	J								
2	1	1				A	C	E	G	N	A	I	J								C2
2	2	1				B	D	F	H	M	B	D	F								C3/C1

Comprehensive PDD Knowledge Base

DS	PG	SubPG	Residual	Order	Occr.	A1	A2	A3	A4	A5	A6	A7	A8	A9	A10	A11	A12	A13	A14	A15	Class
1	1	1	47.16	9	220	A	C	E		M	B	D	F	G							C1
1	1	1	69.78	9	460	A	C		H	M	B	D	F	G							C1
1	1	1	67.64	9	490	A	C	E	H	M		D	F	G							C1
1	1	1	48.34	9	220	A	C	E	H	M	B	D	F								C1
...	...	...	...	...	...	...	...	...	...	...	...	...	...	...							...
1	2	1	53.31	6	1000	B	D	F				I	J								C3
1	2	1	5.38	6	200			F			A	I	J	K							C2
1	2	1	44.24	7	450	B	D	F				I	J	K							C3
1	2	1	39.23	7	630	B	D	F			A	I	J								C3
1	2	1	35.67	8	310	B	D	F			A	I	J	K							C3
...	...	...	...	...	...	...	...	...	...	...	...	...	...	...							...
2	1	1	78.11	8	1000	A	C		G	N	A	I	J								C2
...	...	...	...	...	...	...	...	...	...	...	...	...	...	...							...
2	2	1	30.39	6	370	B	D	F		M	B										C3
2	2	1	20.68	6	370	B		F	H	M	B										C3
2	2	1	26.36	6	510			F	H	M		D	F								C1
...	...	...	...	...	...	...	...	...	...	...	...	...	...	...							...

(e)

Fig. S2-1 Pattern Discovery and Entity Clustering Results of Apriori, PD and PDD. (a) AVA clusters (patterns) for Classes C1, C2 and C3. (b) Pattern samples obtained by Apriori: for support=10%, 4041 patterns were discovered, covering all classes as well as AVs of the random attributes. (c) Pattern samples discovered by traditional PD. There were 12,312 patterns (up to order 10) were discovered. This number could be reduced if we lowered the order of the patterns. (d) Three pattern clusters with 1084, 7758 and 3470 patterns were discovered. Each cluster contains patterns from associating with different or no class labels. Each table shows the AV distributions of the attributes characterizing that cluster. The patterns are subsets of AV combinations in the cluster. (e) PDDKB was obtained by PDD, both Summary PDDKB and Comprehensive PDDKB. It discovers 22 patterns associated with distinct classes as shown. Note that although we allowed patterns up to order 10 to be discovered, the highest order of patterns discovered without relying on class labels was 8.

Thus, this synthetic experimental result demonstrates the necessity of pattern disentanglement of PDD to relate concise/precise patterns to classes. PDD renders a much small set of patterns (only 22) from the DS*s. They are succinctly and accurately associating with the implanted classes, while traditional methods, like Apriori [19] and PD [1] [14], usually produce overwhelming number of overlapping patterns with confused association due to their entanglement in the source environment particularly if the order of patterns specified is high.

Supplementary Results to Analysis I

The PDD experimental results of subclass disentanglement capability is demonstrated in Fig. 2 of the main text. In the Comprehensive PDDKB obtained for APC1 (Fig. 2b), we observed that: a)

the pattern from the SubPG DSU[1 1 2] with 7 occurrences (entities) pertains only to the Mammal-Primate Group (referring to the original data with species' names given, but not shown in this paper); b) the occurrences of the pattern in SubPG DSU[2 2 1] with (S71=M and S72=S) corresponding to Yeast are ten out of twelve; and c) the pattern in SubPG DSU[2 2 3] with S95=A associates only with Fungi. These examples showed that PDD is able to obtain succinct patterns with high precision even from groups with small sample size, supporting the ability of PDD in rendering high precision results.

In Fig. 2b, we observed that the small insect group was identified with the class label given from the third-order pattern (S71=F, S96=N, Class=Insect). However, this pattern disappears in Fig. 2c since it is no more a significant second-order pattern when the class label is dropped because the frequency count required to justify as being a second-order pattern is too low.

Extended PDD results on Breast Cancer and Heart Disease Dataset are given in Fig. S2-2 a, b and c,d respectively. In both sets of results, we observed that the patterns discovered with (Fig. S2-2a, C) or without (Fig. S2-2b,d) class labels given in **R** are almost identical.

From the occurrence frequency of the disentangled patterns, we can locate the rare ones. For example, in Fig. S2-2a, a pattern in SubPG2 of PG1 in DS1 is characterized by cell size=[1 2] that occurs 25 times out of 630 entities. It is a relatively rare. In Fig. S2-2 other patterns of very low frequency could also be found. These are not independent AVs but part of the statistically significant pattern(s). Their occurrences characterized by a unique AV are relatively rare. Their discovery exemplifies PDD capability in handling specific rare cases in precision diagnostics/ therapeutics. In addition, we also observed in Fig. S2-2 that patterns discovered by PDD with or without class label given are identical except that one pattern, with cpt=[3 4] in SubPG2 (Fig. S2-2c) of PG1 in DS1 is not in Fig. S2-2d for the case with no class label given since a single event

cpt=[3,4] alone is not a pattern.

DS1												
PG1	Residual	Occ.	Clump thickness	Cell Size	Cell Shape	Marginal Adhesion	Signle Cell size	Bare Nuclei	Bland	Nucleoli	Mitoses	class
SibPG1	26.18	57	[1 3]	[1 3]	[1 3]	[1 3]			[1 2]	[1 2]		2
	42.74	115	[1 3]	[1 3]	[1 3]	[1 3]	[2 3]	[1 3]		[1 2]		2
	34.78	43	[1 3]	[1 3]	[1 3]	[1 3]	[2 3]	[1 3]	[1 2]	[1 2]		2
SubPG2	4.46	25	[1 3]				[1 2]					
SubPG3	6.54	149	[3 5]	[1 3]								
	5.11	68	[3 5]						[2 3]			
PG2												
SubPG1	269.97	110	[5 10]	[3 10]	[3 10]	[3 10]	[3 10]	[3 10]	[3 10]	[2 10]		4

DS2												
PG1	Residual	Occ.	Clump thickness	Cell Size	Cell Shape	Marginal Adhesion	Signle Cell size	Bare Nuclei	Bland	Nucleoli	Mitoses	class
SibPG1	27.19	355		[1 3]	[1 3]			[1 3]				2
PG2												
SubPG1	50.63	201		[3 10]	[3 10]			[3 10]				4

DS3												
PG1	Residual	Occ.	Clump thickness	Cell Size	Cell Shape	Marginal Adhesion	Signle Cell size	Bare Nuclei	Bland	Nucleoli	Mitoses	class
SubPG1	16.94	314		[1 3]	[1 3]		[2 3]					
PG2												
SubPG1	28.12	217		[3 10]	[3 10]		[3 10]					

(a)

DS1											
PG1	Residual	Occ.	Clump thickness	Cell Size	Cell Shape	Marginal Adhesion	Signle Cell size	Bare Nuclei	Bland	Nucleoli	Mitoses
SubPG1	15.53	61	[1 3]	[1 3]		[1 3]			[1 2]	[1 2]	
	33.42	115	[1 3]	[1 3]	[1 3]	[1 3]	[2 3]	[1 3]		[1 2]	
	27.47	43	[1 3]	[1 3]	[1 3]	[1 3]	[2 3]	[1 3]	[1 2]	[1 2]	
SubPG2	4.46	25	[1 3]				[1 2]				
SubPG3	6.54	149	[3 5]	[1 3]							
	5.11	68	[3 5]						[2 3]		
PG2											
SubPG1	166.68	115	[5 10]	[3 10]	[3 10]	[3 10]	[3 10]	[3 10]	[3 10]	[2 10]	

DS2											
PG1	Residual	Occ.	Clump thickness	Cell Size	Cell Shape	Marginal Adhesion	Signle Cell size	Bare Nuclei	Bland	Nucleoli	Mitoses
SibPG1	16.94	314		[1 3]	[1 3]		[2 3]				
PG2											
SubPG1	28.12	217		[3 10]	[3 10]		[3 10]				

DS3											
PG1	Residual	Occ.	Clump thickness	Cell Size	Cell Shape	Marginal Adhesion	Signle Cell size	Bare Nuclei	Bland	Nucleoli	Mitoses
SubPG1	24.38	324		[1 3]	[1 3]			[1 3]		[1 2]	
PG2											
SubPG1	27.99	211		[3 10]	[3 10]			[3 10]			

(b)

DS1																
**PG1**	**Residual**	**Occ.**	**age**	**sex**	**cpt**	**rbp**	**sc**	**fbs**	**rer**	**mhra**	**eia**	**oldpeak**	**spess**	**nmvc**	**thal**	**class**
**SubPG1**	24.58	19	[29 51]							[162 202]	0	[0 0.1]	1	[0 1]	3	**1**
	21.87	8	[29 51]		[1 3]					[162 202]	0	[0 0.1]	1	[0 1]	3	**1**
**SubPG2**	4.88	62			[3 4]											**1**
**PG2**	**Residual**	**Occ.**	**age**	**sex**	**cpt**	**rbp**	**sc**	**fbs**	**rer**	**mhra**	**eia**	**oldpeak**	**spess**	**nmvc**	**thal**	**class**
**SubPG1**	12.05	38			[4 4]					[71 143]		[1.4 6.2]				**2**
	14.52	15	[59 77]							[71 143]		[1.4 6.2]	2	[1 3]		**2**
	12.67	16	[59 77]		[4 4]							[1.4 6.2]	2	[1 3]		**2**
	32.06	13			[4 4]					[71 143]	1	[1.4 6.2]	2	[1 3]	7	**2**
	24.88	6	[59 77]		[4 4]					[71 143]	1	[1.4 6.2]	2	[1 3]	7	**2**

(c)

DS1															
**PG1**	**Residual**	**Occ.**	**age**	**sex**	**cpt**	**rbp**	**sc**	**fbs**	**rer**	**mhra**	**eia**	**oldpeak**	**spess**	**nmvc**	**thal**
**SUbPG1**	17.88	19	[29 51]							[162 202]	0	[0 0.1]	1	[0 1]	3
	16.1	8	[29 51]		[1 3]					[162 202]	0	[0 0.1]	1	[0 1]	3
**PG2**	**Residual**	**Occ.**	**age**	**sex**	**cpt**	**rbp**	**sc**	**fbs**	**rer**	**mhra**	**eia**	**oldpeak**	**spess**	**nmvc**	**thal**
**SubPG1**	7.06	16	[59 77]		[4 4]								2	[1 3]	7
	8.44	17	[59 77]		[4 4]					[71 143]			2	[1 3]	
	7.35	14	[59 77]		[4 4]					[71 143]		[1.4 6.2]	2		
	9.24	16	[59 77]		[4 4]					[71 143]		[1.4 6.2]		[1 3]	
	21.04	13			[4 4]					[71 143]	1	[1.4 6.2]	2	[1 3]	7
	16.41	6	[59 77]		[4 4]					[71 143]	1	[1.4 6.2]	2	[1 3]	7

(d)

Fig. S2-2 Results of Pattern Discovery and Disentanglement on Breast Cancer and Heart Disease Datasets. (a) Discovered Patterns for Cancer Dataset with class label given in **R** and (b) class label not given in **R.** (c) Discovered Patterns for Heart Disease Dataset with class label given in **R** and (d) class label not given in **R.**

Analysis II: Unsupervised Learning Performance

In the main text (Fig. 3) we see the superior clustering performance of PDD over K-Mean on the APC of Class A Scavenger Receptors. Here, using this set of data, we will show the noise tolerance capability of PDD in comparison with its counterparts (Fig. 2-3 a and b). Next, we also supplement the comparative clustering results obtained from the Breast Cancer and the Heart Disease datasets (Fig. 2-3 c and d).

To test the noise tolerance performance of PDD, the column noises were added into the original clean and succinct APC1 from three columns to fifteen columns. We then compared the clustering scores: Accuracy, Precision, Recall, F-measure on Breast Cancer and Heart Disease Dataset based on the given ground truth [9]. As shown in Fig. S2-3a and b, both K-means and PDD could obtain high clustering accuracy even without class information for the original APC1 dataset. However, after adding background noise to the APCs (with three to fifteen noise columns), the accuracy of

K-means was reduced whereas that of PDD remained essentially no change. This further validates the robustness of PDD against noise. Besides, K-means could not render explicit displayable patterns/knowledge inherent in each cluster, whereas PDD could. This also validates that PDD does not rely on feature engineering yet possesses the ability to unveil interpretable feature association at a deeper AVA level.

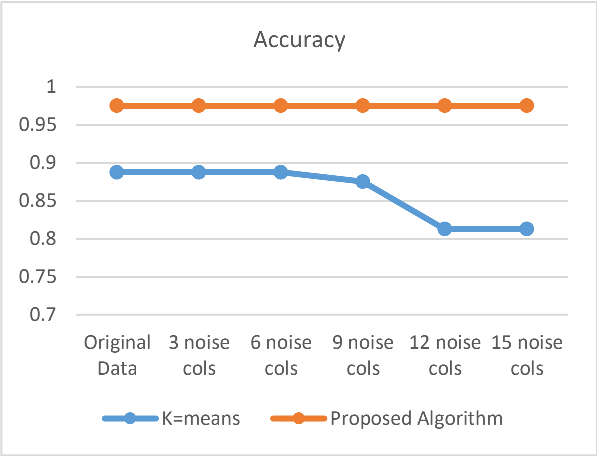

(a) Clustering Accuracy on APC Dataset with Noise

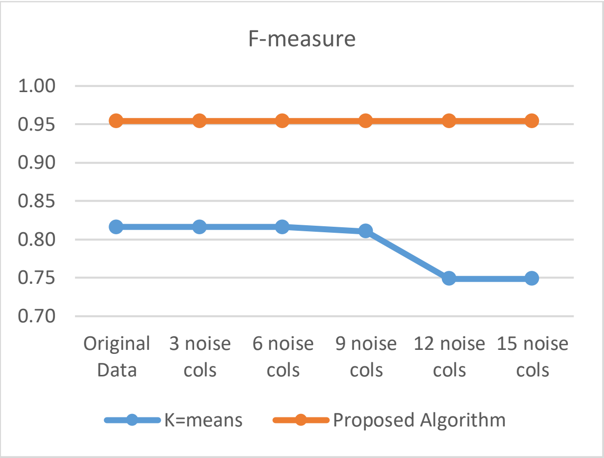

(b) F-measure on APC Dataset with Noise

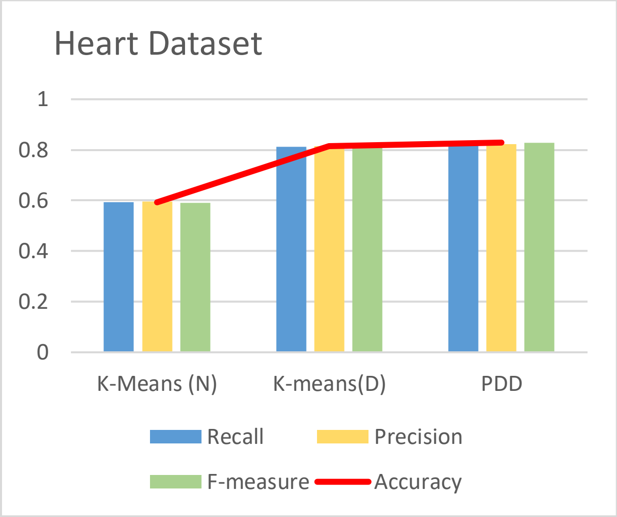

(c) Clustering Results on Heart Dataset

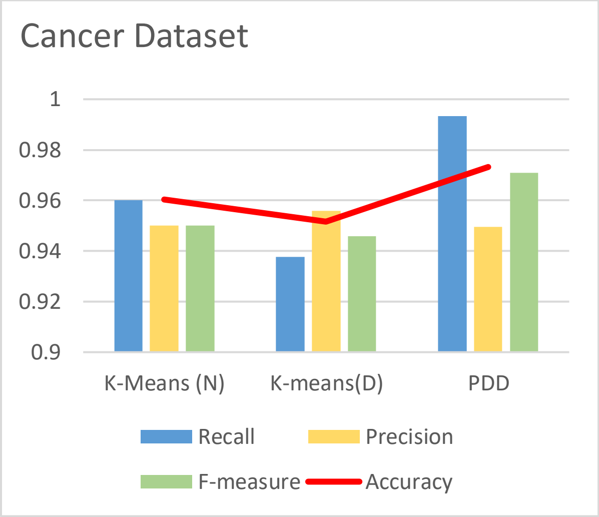

(d) Clustering Results on Cancer Dataset

Fig. S2-3 Results of Entity Clustering. (a) and (b): Comparison of Segment Clustering Result on Cytochrome c APC Dataset with Noise Attributes (noise columns in **R**) added. (c) The comparison of entity clustering results on Breast Cancer Dataset of K-means (on numerical data (N) and discrete data (D)) and PDD and (d) that on Heart Dataset results of K-means and PDD.

In addition, Fig. S2-3c, d showed the comparison clustering results for the Heart and the Cancer datasets respectively with no noise added. For Heart Disease, we noted from Fig. S2-3c shows that that PDD (with F-measure=0.83, Accuracy=82.87%) outperformed K-Means on both original numerical (F-measure=0.59, Accuracy=59.26%) and discretized datasets (F-measure=0.81, Accuracy=81.48%) in F-measure and Accuracy. For Cancer, Fig. S2-3d shows the results of Accuracy and F-measure of PDD vs K-Means on the discretized datasets are closer since this dataset contains less noise. But the leverage is that PDD could reveal all the patterns in the Entity Clusters while K-Means could not. It opens the door to visualize patterns in clusters formed.

Analysis III: Mislabels Detection Using Unsupervised Learning

To solve this problem, PDD uses the knowledge it acquires in the PDDKB. Fig. S2-4a and S2-4b show the PDDKB obtained from APC1 and Heart respectively. Each contains the DS, Pattern and Entity sections (Table 1, main text). Summary Patterns (SP) and the Comprehensive set of Patterns (CP) discovered in the DSUs. SP helps to interpret the functionality implicitly associated with some of the high-order patterns that make up the super-pattern in each DSU.

In the APC1 case (Fig. S2-4a), there are 80 entities in total. The first 30 entities (columns) belong to class "Mammal" (colored by blue in Fig. S2-3a), and entities 21-55 belong to "Plant" (orange), entities 56-75 to "Fungus" (yellow), and the last five to Insect (green). We also observed that the entities E76 and E80 also share the subpattern (- - E E I A - -) of the Mammal group (first row of the Comprehensive PDDKB in Fig. S2-4A). This also showed that certain insects shared this subpattern with the Mammal Group in this APC as confirmed by a close look at the data. This shows that while PDD is able to discover the dominating pattern succinctly pertaining to the taxonomical classes, it does not miss the sub-pattern shared by some cases of these two species.

In this PDDKB, for most entities, correct taxonomic classes found by PDD are revealed (Fig. S2-4). Meanwhile, PDD also unveils outliers and mislabeled entities such as the two entities E55 and E66 respectively, both of which are enclosed in the red box (Fig S2-4a). E55, the sample of the plant kelp, is an outlier since its pattern is not sharing statistically significant patterns of other groups. E66 is a mislabeled entity since it was identified by PDD as "Plant" since it shared two significant patterns in the plant group but none in the fungus group, but it was labelled as "Fungus" in the original data. Hence, we conjecture that E55 is an outlier and E66 is a mislabeled entity.

Similarly, the analysis results for Heart Disease are given in Fig. S2-4b. For most entities, correct classes complying with class labels in **R** inserted after clustering were found. Moreover, like in the APC1 case, PDD unveiled two mislabeled cases (red boxes in Fig. S2-4b), E122 and E131. They contain patterns associated with "Absence" of heart disease but were labeled as "Presence".

All these cases show that if outliers and mislabeled entities exist in a dataset with class labels given, PDD can spot them without the requirement of additional process. This is very important in real world application, particularly in healthcare, where rare cases or patients in the early stage of a disease can be spotted.

Summary PDD Knowledge Base

DS				Patterns									Entities																		
													Mammal					Plant				Fungi							Insect		
DS	PG	Sub PG	class	S71	S72	S73	S76	S88	S90	S92	S95	S96	1	2	3	...	30	31	32	54	55	56	57	...	66	...	75	76	...	80
1	1	1	Mammal		M	E	E	I	A	I	K	G	3	4	3		4											1		1
1	1	2	Mammal		M			I	V	I	K	E																		
1	2	1	Plant		Y	D	L	V	P	L	P	Q						1	1	1					2					
2	1	1	Plant		Y		L	V	P			Q						1	1	1										
2	2	1	Fungi	M	S/F			A	G		E	K										2	2				3			
2	2	2	Fungi		F																									
2	2	3	Fungi		S			A	G		A	K																		
3	1	1	Insect		F							N																1		1

Comprehensive PDD Knowledge Base

PC	PG	Sub PG	Residual	Order	Occr.	class	S71	S72	S73	S76	S88	S90	S92	S95	S96	1	2	3	...	30	31	...	54	55	56	57	...	66	...	75	76	...	80
1	1	1	10.36	4	27				E	E	I	A				1	1	1	...	1		...					...		...		1	...	1
1	1	1	52.44	7	25	Mammal		M	E	E	I		I	K		1	1	1	...	1		...					...		...			...	
...	...		...	...	...	....	...	...	...	...	...	...	...	...	...																		
1	2	1	218.97	9	19	Plant		Y	D	L	V	P	L	P	Q				...		1	...	1				...		...			...	
...	...		...	...	...	....	...	...	...	...	...	...	...	...	...																		
2	1	1	81.64	6	21	Plant		Y		L	V	P			Q				...		1	...	1				...		...			...	
...	...		...	...	...	....	...	...	...	...	...	...	...	...	...																		
2	2	1	32.55	4	18	Fungi					A	G			K				...			...			1	1	...		...	1		...	
2	2	1	139.93	6	10	Fungi	M	S			A	G			K				...			...					...		...	1		...	
2	2	1	251.05	7	5	Fungi	M	S			A	G		E	K				...			...					...		...	1		...	
2	2	2	2.62	2	7	Fungi		F											...			...			1	1	...		...			...	
...	...		...	...	...	....	...	...	...	...	...	...	...	...	...																		
3	1	1	20.02	3	5	Insect		F							N				...			...					...		...		1	...	1

(a)

Summary PDD Knowledge Base

DS				Summary Patterns													Entities									
Disease Complex/Class				sign/symptoms/lab tests													Absence					Presence				
DS	PG	SubPG	class	age	sex	cpt	rbp	sc	fbs	rer	mhra	eia	oldpeak	spess	nmvc	thal	1	2	...	122	131	151	152	...	269	270
1	1	1	Absence	[29 51]	F	2;3					[162 202]	0	[0 0.1]	1	[0 1]	3	33	6	...					...		
1	2	1	Presence	[59 77]	M	4					[71 143]	1	[1.4 6.2]	2	[1 3]	7			...	1	4	45	3	...	5	66
2	1	1	Presence			4								2	[1 3]	7			...			7	1	...	1	7
2	2	1	Absence											1	[0 1]	3	5	2	...					...		
PDD disentangles the dataset into two DSs. In DS1, two Pattern Groups are discovered for Absence and Presence. In DS2, two pattern groups are discovered with low-order patterns.				Dataset contains 13 mixed-mode attributes (i.e. Real, Ordered, Binary, Nominal). Each row represent a summary patterns (Attribute Cluster). e.g. The first row represents in DS1 and PG1, the attribute cluster contains 11 attribute values (class=Absence; age=[29 51]; sex=F; cpt=2/3; mhra=[162 202]; ... ... thal=3.)													270 entities in Heart Disease data set Entities 1-150: Absence; 151-270: Presence The value in the block, such as 33, means there are 33 high-order patterns are grown from the first AV Cluster covered by the first entity. Each of these patterns will be displayed in the comprehensive PDDKB									

Comprehensive PDD Knowledge Base

DS	PG	SubPG	Residual	Order	Occr.	class	age	sex	cpt	rbp	sc	fbs	rer	mhra	eia	oldpeak	spess	nmvc	thal	1	2	...	150	151	...	269	270
1	1	1	6.87	3	103	Absence									0				3	1	1	...			...		
1	1	1	9.18	4	73	Absence									0		1		3	1	1	...			...		
1	1	1	9.3	4	57	Absence		F							0				3		1	...			...		
1	1	1	7.37	4	46	Absence			3						0				3	1		...			...		
1	1	1	9.43	5	27	Absence		F	3						0				3			...			...		
1	1	1	9.94	5	43	Absence		F							0			[0 1]	3			...			...		
...	...	...	...	...	...	...	...	...	...	...	...	...	...	...	...	...	...	...	...	...	...	...	...	...	...	...	...
1	2	1	6.88	3	68	Presence		M											7			...			...	1	
1	2	1	4.77	3	64	Presence		M									2					...		1	...		1
1	2	1	4.36	3	38	Presence	[59 77]										2					...		1	...		1
1	2	1	6.57	3	58	Presence			4								2					...		1	...		1
...	...	...	...	...	...	...	...	...	...	...	...	...	...	...	...	...	...	...	...	...	...	...	...	...	...	...	...

(b)

Fig. S2-4 Summary PDDKB and Comprehensive PDDKB (a)Result for APC1 (b)Result for Heart Disease Data Set

Analysis IV: Classification/Prediction Assessment and Comparison

In Analysis IV, the comparison of classification results between PDD and other classification methods were provided. In all our experiments, 80% of the available data for each class was

selected randomly as training data and the 20% remaining was retained as testing data. We compared the results obtained from PDD with those from Support Vector Machine (SVM) and Artificial Neural Network (ANN) [20], first using the original dataset and then the dataset after the outliers and mislabeled entities were removed. The experimental runs were iterated 10 folds to calculate the average classification accuracy for performance assessment and comparison.

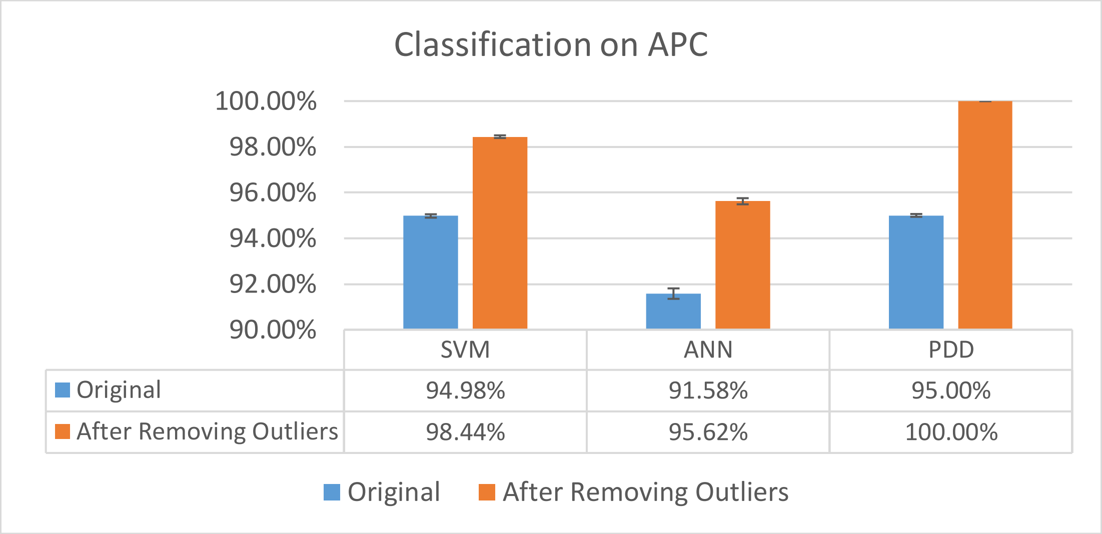

(a)

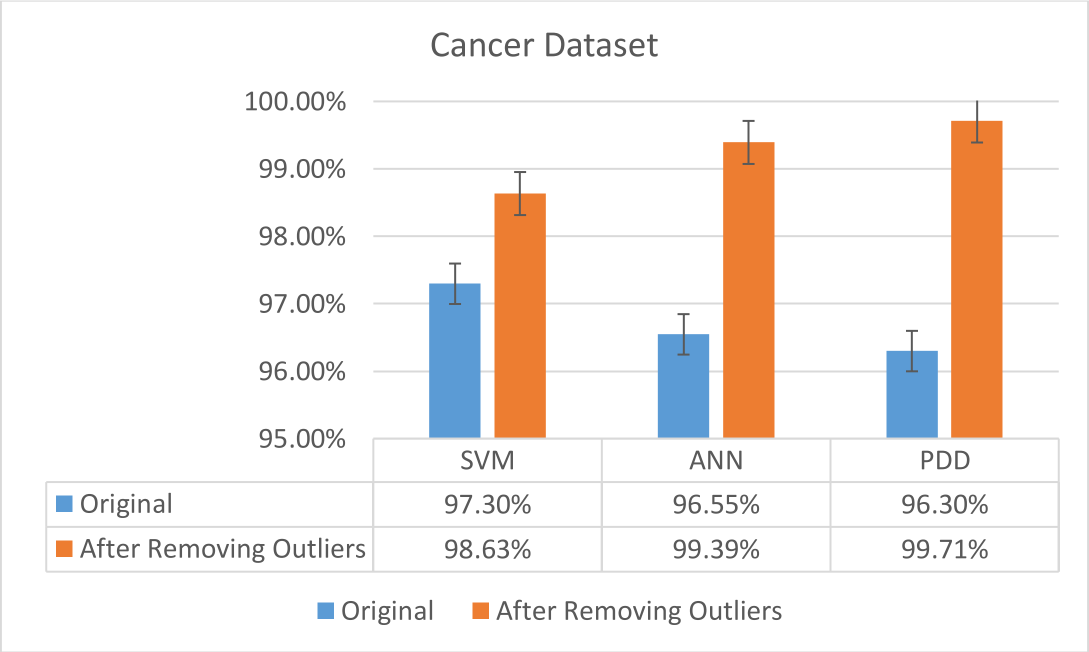

(b)

Fig. S2-5 Comparison of Classification Result between Original Dataset and Dataset after Removing Outliers (a) on APC1 Dataset (b) on Breast Cancer Data Set

For APC1, Fig. S2-5a shows the comparison result of the classification accuracy with variance for the three ML models. We found that, after removing the outliers and mislabeled cases, the classification results obtained from each of these algorithms were improved approximately 5%. Fig. S2-5b shows the classification accuracy with variance by these different algorithms for Breast Cancer dataset. Again, we found approximate 5% -10% improvement on these datasets respectively after removing the outliers and mislabeled entities identified by PDD. We should note that the percentage of improvement depends on the size of the anomalies found from the original dataset before training.

Supplement Note 3: Related Work

Deep Learning and PDD

Deep learning (DL) is a particular method of ML. It is a powerful learning tool for tasks related to images, signals, speech and text, where humans can cognitively relate inputs to outputs. By this token, successful analyses/classifications on medical scans, X-rays, retinal images, ECG, etc. have been reported. However, in more general healthcare data analytics based predominantly on clinically recorded data with numeral and descriptive features, the input and output relations are not that obvious, particularly when the correlation of signs, symptoms, test results of the patients could be the manifestation of multiple factors. Thus, it is very difficult to apply DL to solve such problems with given features. Hence, this poses a challenge to DL in clinical application. However, PDD provides succinct and explicit ways to relate the patient patterns discovered from their heath records and symptomatic patterns to the etiological causes/classes, patient groups and individuals. It displays such comprehensive explainable knowledge in the PDDKB.

Another concern of DL in data analytics is on the transparency and assured accuracy [21] [22]. As for transparency, DL is generally considered as a black box [23]. As for assured accuracy, DL usually requires large size of data with a broad base of coverage and experts' supervision to ensure high predictive accuracy. Without such assurance, sometimes researchers found that simpler, cheaper and more useful data modeling could render better results [21]. PDD is able to overcome these two problems. It can reveal and evaluate the inputs, throughputs and output in an explicit manner. It can spot errors, disentangle and select entangled AVAs to render accountable and succinct results to ensure trust, accountability and assurance. Furthermore, it also resolves another issue confronting DL, that is the lack of ability to discern the correctness/robustness of the ground

truth usually taken for granted from the given data. Thus, it largely relies on human guidance or presumed prior knowledge and the use of large data to overwhelm the minor errors while the errors are still there. However, PDD provides a novel way to identify and remove the anomalies and mislabels instead of suppressing them by using more data. Through disentanglement and significant statistic screening and testing to render robust/relevant patterns, PDD attains explainability and assurance while DL finds it difficult to attain.

In summary, when applying to precision medicine and discovering rare cases, DL still requires strong support of prior knowledge and feature engineering [24] which is very difficult and demanding enormous human effort. While the medical community still wishes to understand how machines learn [25] and what sort/level of accountable knowledge they could offer, they are looking for an AI system which is explainable and accountable, able to discover anomalies and rare cases [21] [26] without sacrificing predictive accuracy. This is what PDD can offer. While empirical science relies on statistics, PDD renders deeper statistical patterns [12] and ensures the robust use of statistics in scientific exploration. It attempts to provide solutions to the above problems confronting DL and relational data analysis.

Related work in Frequency Pattern Mining and Pattern Discovery

To render transparency and interpretability, Decision Trees and Forests, Frequent Pattern Mining or Pattern Discovery were proposed. For decades, *Frequent Pattern Mining* [27] [19] [1] is an essential data mining task to discover knowledge in the form of association rules from relational data [1]. Most of them are based on the likelihood, weight of evidence [1], support, confidence and/or statistical residuals [19] [1]. However, as revealed in our recent work, [4] [3] [2], associations discovered from relational data could be entangled due to multiple entwining functional characteristics inherent in the source environments. Hence, the patterns discovered

directly from the acquired data may have overlapping or functionally entwined attribute values as observed from our recent works [4] [2]. This notion is further validated and exemplified by our synthetic experiment in S2, resulting in serious pattern overlapping/redundancy [8] [14] and uncontrollable entwinement. These overwhelming yet statistically legitimate patterns are difficult to partition and summarize [8] [14] [6]. Hence, patterns discovered by current pattern mining and discovery methods are unable to reveal and use the deep knowledge inherent in the data without pattern disentanglement.

In the past decades, many methods [19] have been developed for discovering high-order patterns or mining frequent itemsets/rules [27] [1]. However, their performance is sensitive to manually set thresholds such as support and confidence and the patterns they discovered were overwhelmed [14] with overlapping/redundant patterns due to their combinatorial nature and the possible entanglement in the source environment. Optimal ways of identifying, partitioning and grouping patterns were lacking. Pattern clustering [14] attempts to group them for better visualization and interpretation. However, typically they still produce too many pattern clusters and cluster configurations. Although our later work, such as pattern pruning and summarization [14] [8], attempted to produce more succinct representation when similar patterns are clustered into groups, such approaches still require intensive search, and it is hard to find effective similarity measures to direct the clustering. After a long search for better optimal methods, we later found that the key problem lies in the entanglement of AVA due to multiple governing functions/factors in the source environment [3]. Most of the previous methods cannot discriminate the subtle variations inherent in the entangled sources. Such observation poses a challenge to current DL/ML models since, in these cases, the input-to-output relationship they are based upon, if not properly disentangled, is not succinct to render good solutions. Hence, in the problem as revealed in recent study, we found

that both the above-mentioned approaches lack effective ways to disentangle the statistics obtained from the data coming from subtle multiple entangled sources.

Furthermore, we also notice that existing ML approaches are still encountering difficult problems concerning transparency, low data volume, imbalance classes and presence of anomalies, bias and mislabeled samples [21] [26]. Hence, PDD was developed to meet these challenges.

Aligned Pattern Clusters (APCs) and the Related Work

Biomolecules are coded for intriguing structure/function operating in complex physicochemical environments. Functional region identification is of fundamental importance for protein/DNA/RNA sequences analysis. Such knowledge provides better scientific understanding and could assist drug discovery and precision medicine. Aligned Pattern Clusters (APCs) [14] [5] [28] were introduced to reveal conserved functional regions with variable width from protein family sequences. The significance of APC is due to its dual space representation, consisting of the pattern and the data space. The former displays the aligned patterns with statistical significance supports (the "what"); the latter displays all the patterns in the APC on the original sequence space, the "where" and the delimited range of the domain covering all its patterns. Our Aligned Pattern Clustering Algorithm (APCn) [5] [28] is able to discover aligned patterns even with minor substitution and insertion/deletion mutations. Hence it is able to support genomic and proteomic research [29] and precision medicine when the mutation spots could be rare [28].

APCn has broad applications in genomics, drug discovery and precision medicine [5] [28] [30] [29]. In the early days, we obtained from DNA three pattern clusters corresponding to three types of splicing-junctions: exon/intron (EI boundaries), intron/exon (IE boundaries), and Neither. We could use the discovered patterns to classify segments pertaining to these junctions [14]. Later we developed APCn to reveal protein functionality of bio-sequence families [5] [29] [4] [2]via their

conserved patterns. When applied to the cytochrome c, the ubiquitin, and the triosephosphate isomerase (TIM) protein families, the APCs discovered were related to binding segments, sites and residues [5] [31]. Furthermore, we were able to discover Protein-DNA binding cores obtained from the Cloud [30]. Our algorithms toke protein and DNA sequences from TRANSFAC (a Protein-DNA Binding Database) as input; discovered from both sets of sequences conserved regions via APCs discovered; associated them as Protein-DNA Co-Occurring APC pairs; and found, among the top co-occurring pairs, the 3-dimensional structures from Protein Data Bank (PDB) to validate the binding core found. If no 3-D model was found, homology modeling was applied to their close matches in PDB to attain new chemically feasible binding cores. Our algorithm obtained binding cores with higher precision and much faster runtime (_1600x) than that of its contemporaries [32].

Using known and putative sequences of two proteins belonging to a relatively uncharacterized protein family we were able to group evolutionarily related sequences and identified conserved regions [32]. Furthermore, combining our data and class measures allowed us to interpret the results by inferring regions of biological importance within the binding domain of these proteins. As an unsupervised tool [33], APCn was able to discover and analyze patterns in bio-sequences for 1) identification of protein binding sites, 2) revealing functioning subgroup characteristics through PDD [2] [4], and 3) identification of intra-protein, inter-protein and protein-DNA binding cores. From the related work, we have shown the significance of APCs in revealing subtle classes, and functional domains. Hence, we choose APCs as relational models to assess PDD's capability in revealing patterns, pattern subgroups, pattern characteristics relating functional domains, rare classes/groups/mutations and hotspots in bio-sequences which contain crucial information for precision medicine. In this paper, we present PDD's capability on solving this type of data and problems.

References

[1] A. K. Wong and Y. Wang, "High-Order Pattern Discovery from Discrete-Valued Data," *IEEE Transaction On Knowledge System,* vol. 9, no. 6, pp. 877-893, 1997.

[2] P.-Y. Zhou, A. Sze-To and A. K. Wong, "Discovery and disentanglement of aligned residue associations from aligned pattern clusters to reveal subgroup characteristics," *BMC medical genomics,* vol. 11, no. 5, p. 103, 2018.

[3] A. K. Wong, A. H. Y. Sze-To and G. L. Johanning, "Pattern to Knowledge: Deep Knowledge-Directed Machine Learning for Residue-Residue Interaction Prediction," *Nature Scientific Reports,* vol. 8, no. 1, pp. 2045-2322, 2018.

[4] P.-Y. Zhou, A. E. Lee, A. Sze-To and A. K. Wong, "Revealing Subtle Functional Subgroups in Class A Scavenger Receptors by Pattern Discovery and Disentanglement of Aligned Pattern Clusters," *Proteomes,* vol. 6, no. 1, p. 10, 2018.

[5] A. K. Wong and A. E. Lee, "Aligning and clustering patterns to reveal the protein functionality of sequences," *IEEE/ACM Transactions on Computational Biology and Bioinformatics (TCBB),* vol. 11, no. 3, pp. 548-560, 2014.

[6] J. Cheng, Y. Ke and W. Ng, "\delta-Tolerance Closed Frequent Itemsets," in *Data Mining, 2006. ICDM'06. Sixth International Conference on. IEEE*, 2006.

[7] J. LI, G. Liu and L. Wong, "Mining statistically important equivalence classes and delta-discriminative emerging patterns," in *Proceedings of the 13th ACM SIGKDD international conference on Knowledge discovery and data mining. ACM*, 2007.

[8] P.-Y. Zhou, G. C. Li and A. K. Wong, "An Effective Pattern Pruning and Summarization Method Retaining High Quality Patterns With High Area Coverage in Relational Datasets," *IEEE Access,* vol. 4, pp. 7847-7858, 2016.

[9] N. Abdelhamid and F. Thabtah, "Associative classification approaches: review and comparison," *Journal of Information & Knowledge Management,* vol. 13, no. 03, p. 1450027, 2014.

[10] G. Cumming, Understanding the new statistics: Effect sizes, confidence intervals, and meta-analysis, Routledge, 2013.

[11] T. Elgamal and H. Mohamed , "Analysis of PCA algorithms in distributed environments," 13 5 2015. [Online]. Available: https://arxiv.org/abs/1503.05214. [Accessed 17 3 2015].

[12] A. Singh and V. Sarjolta, "MapReduce WordCount: Execution and Effects of Altering Parameters," *Int. J. Innov. Res. Comput. Commun. Eng.,* vol. 3, no. 10, p. 9330–9336, 2015.

[13] P.-N. Tan, M. Steinbach, A. Karpatne and V. Kumar, Introduction to data mining, New York: Pearson Education, 2018, pp. 327-414.

[14] A. K. Wong and G. C. Li, "Simultaneous pattern and data clustering for pattern cluster analysis," *IEEE Transactions on Knowledge and Data Engineering,* vol. 20, no. 7, pp. 977-923, 2008.

[15] F. Whelan, C. Meehan, G. B. Golding, B. McConkey and D. M. Bowdish, "The evolution of the class A scavenger receptors.," *BMC evolutionary biology,* vol. 12, no. 1, p. 227, 2012.

[16] W. H. Wolberg, "Breast Cancer Wisconsin (Original) Data Set," [Online]. Available: https://archive.ics.uci.edu/ml/datasets/breast+cancer+wisconsin+(original).

[17] A. Asuncion and D. Newman, "UCI Machine Learning Repository," School of Information and Computer Science,University of California, Irvine, CA, 2007. [Online]. Available: http://archive.ics.uci.edu/ml/.

[18] "Statlog (Heart) Data Set," [Online]. Available: https://archive.ics.uci.edu/ml/datasets/Statlog+(Heart).

[19] C. C. Aggarwal and J. Han, Frequent pattern mining, Springer, 2014.

[20] S. S. NIKAM, "A comparative study of classification techniques in data mining algorithms," *Oriental journal of computer science & technology,* vol. 8, no. 1, pp. 13-19, 2015.

[21] E. J. Topol, "High-performance medicine: the convergence of human and artificial intelligence," *Nature medicine,* vol. 25, no. 1, pp. 44-56, 2019.

[22] W. Samek, T. Wiegand and K. Müller, "Explainable artificial intelligence: Understanding, visualizing and interpreting deep learning models," *arXiv preprint arXiv:1708.08296,* 2017.

[23] P. Voosen, "How AI detectives are cracking open the black box of deep learning," *Science,* 2017.

[24] D. Chen, S. Liu, P. Kingsbury, S. Sohn, C. B. Storlie, E. B. Habermann, J. M. Naessens, D. W. Larson and H. Liu, "Deep learning and alternative learning strategies for retrospective real-world clinical data," *npj Digital Medicine,* vol. 2, no. 1, p. 43, 2019.

[25] D. Castelvecchi, "Can we open the black box of AI?," *Nature News,* vol. 538, no. 7623, p. 20, 2016.

[26] C. Aggarwal and S. Sathe, "Bias Reduction in Outlier Ensembles: The Guessing Game," in *Outlier Ensembles*, Springer, 2017.

[27] S. Naulaerts, W. Bittremieux, T. Vu, W. Vanden Berghe, B. Goethals and K. Laukens, "A Primer to frequent itemset mining for bioinformatics," *Briefings in bioinformatics,* vol. 16, no. 2, pp. 216-231, 2015.

[28] A. H. Szeto and A. K. Wong, "Discovering Patterns from Sequences Using Pattern-Directed Aligned Pattern Clustering," *IEEE Trans. On Nanobioscience,* vol. 14, no. 8, 2018.

[29] S. Han, P. Bharadwaj, K. Novakowski, A. Lee, A. Wong and D. Bowdish, "Pattern associated modelling for discovery of novel protein motifs in the macrophage scavenger receptors (IRM5P.703)," *The Journal of Immunology,* vol. 62, no. 4, p. 192, 2014.

[30] E. Lee, H. Sze-To, M. Wong, K. Leung, T. Lau and A. Wong, "Discovering protein-dna binding cores by aligned pattern clustering," *IEEE/ACM transactions on computational biology and bioinformatics,* vol. 14, no. 2, pp. 254-263, 2015.

[31] A. E.-S. Lee, S. Fung, H. Sze-To and A. Wong, "Discovering co-occurring patterns and their biological significance in protein families.," *BMC bioinformatics,* vol. 15, no. 12, p. S2, 2014.

[32] T. Chan, L. Lo, H. Sze-To, K. Leung, X. Xiao and M. Wong, "Modeling associated protein-DNA pattern discovery with unified scores," *IEEE/ACM transactions on computational biology and bioinformatics,* vol. 10, no. 3, pp. 696-707, 2013.

[33] E. Lee, H. Sze-To, A. Wong and D. Stashuk, "Unsupervised Pattern Discovery in Biosequences Using Aligned Pattern Clustering," *SM Journal of Bioinformatics and Proteomics,* vol. 1, no. 2, p. 1008, 2016.

[34] E. Lee, F. Whelan, D. Bowdish and A. Wong, "Partitioning and correlating subgroup characteristics from Aligned Pattern Clusters," *Bioinformatics,* vol. 32, no. 16, pp. 2427-2434, 2016.

[35] E. Lee, A. Sze-To, A. Wong and D. Stashuk, "Unsupervised Pattern Discovery in Biosequences Using Aligned Pattern Clustering.," *SM J Bioinform Proteomics,* vol. 1, no. 2, p. 1008, 2016.
